# Supplementary material for: Investigating the effectiveness of care delivery at an acute geriatric community hospital for older adults in the Netherlands: a protocol for a prospective controlled observational study
Source: BMJ Open. 2020 Mar 31;10(3):e033802. doi: 10.1136/bmjopen-2019-033802 (PMC7170597; doi:10.1136/bmjopen-2019-033802)
Supplement: Supplementary data [file bmjopen-2019-033802supp002.pdf]

Supplementary table Overview of the content and description of (outcome) measurements and timing of measurements at the Acute Geriatric Community Hospital compared to the measurements available in the two control groups.

	**Description and/or instrument**	**H1**	**H2**	**P1**	**P3**	**P6**
1. Medical and demographical data						
**Sociodemographic data**	Date of birth, age at admission, sex, level of education, living conditions, marital status	T A				
**Data on admission**	Time spent at the ED*, admission diagnosis, date and time of admission	T A				
**Chronic conditions**	Charlson Comorbidity Index[38]	T A				
**Polypharmacy**	Number of drugs	T A				
**Mortality**	Date of death		T A			
2. Cognitive functioning						
**Cognitive impairment**	Mini Mental State Examination (MMSE)	T A				
**Delirium**	Safety management system patient screening (VMS)[41]<br>Confusion Assessment Method (CAM)[39]<br>Delirium Observation Scale (DOS)[42]	T<br>TA†<br>T†	 <br>T			
3. Psychosocial functioning and quality of life						
**Apathy**	Geriatric Depression Scale (GDS-3)[43]	A	A	A	A	
**Social network and informal care**	Presence and frequency of informal care	T		-	-	T
**Quality of life and health status**	EQ-5D[36]	T A		A	A	T A
4. Physical functioning						
**Identifying at-risk-patients**	ISAR-HP- Identifying Seniors at Risk score[44]	T				
**Functional status**	Activities of daily Living (ADL) modified Katz-ADL score[35]	T A	A	A	A	T
**(Im)mobility**	Using walking aid, information from the Katz-ADL questions on exercise	T A				
**Handgrip strength**	Jamar[48]	T A	A			
**Gait speed**	Short Physical Performance Battery SPPB[49]	T A	A			
**Falling**	Fall history<br>Falls in the AGCH<br>Numeric Rating Scale (NRS) on the fear of falling[34]	T A<br>n/a<br>A	-<br>n/a<br>A	A<br>n/a<br>A	A<br>n/a<br>A	T<br>n/a<br>-
**Pain**	Numeric Rating Scale (NRS) on pain[50]	T A	A	A	A	-
**Fatigue**	Numeric Rating Scale (NRS) on fatigue[51]	T A	A	A	A	-
**Nutrition**	Short Nutritional Assessment Questionnaire (SNAQ)[52]	T A				
5. Healthcare utilization and satisfaction with care						
**Medical care during admission**	Diagnostics performed in the AGCH<br>Readmission to university hospital		n/a			

	Length of stay at the AGCH					
**Hospital readmission**	Readmission rate to the hospital or AGCH			A	T A	T
**Health care utilization**	Home care, medical specialist care, temporary institutional care, primary care.	T		A	A	T
**Satisfaction with Care**	Eight question questionnaire[53]		-	-		

Grey tone= measurement in prospective cohort study at the AGCH.

H1= at admission, H2= at discharge, P1= one month after discharge, P3 = three months after discharge, P6 = six months after discharge.

T= available from *Transitional Care Bridge study(TCB)*[1]

A= available from *Hospital- ADL study(H-ADL)*[2]

** and* - =Not available from TCB or H-ADL

†=Single baseline measurement

n.a.= not applicable
