# Supplementary material for: Investigating the effectiveness of care delivery at an acute geriatric community hospital for older adults in the Netherlands: a protocol for a prospective controlled observational study
Source: BMJ Open. 2020 Mar 31;10(3):e033802. doi: 10.1136/bmjopen-2019-033802 (PMC7170597; doi:10.1136/bmjopen-2019-033802)
Supplement: Author's manuscript [file bmjopen-2019-033802.draft_revisions.pdf]

BMJ Open

BMJ Open is committed to open peer review. As part of this commitment we make the peer review history of every article we publish publicly available.

When an article is published we post the peer reviewers' comments and the authors' responses online. We also post the versions of the paper that were used during peer review. These are the versions that the peer review comments apply to.

The versions of the paper that follow are the versions that were submitted during the peer review process. They are not the versions of record or the final published versions. They should not be cited or distributed as the published version of this manuscript.

BMJ Open is an open access journal and the full, final, typeset and author-corrected version of record of the manuscript is available on our site with no access controls, subscription charges or pay-per-view fees (http://bmjopen.bmj.com).

If you have any questions on BMJ Open's open peer review process please email info.bmjopen@bmj.com

BMJ Open

Evaluating an acute geriatric community hospital for older adults in a prospective cohort study compared with two historical control groups: a study protocol

Journal:	*BMJ Open*
Manuscript ID	bmjopen-2019-033802
Article Type:	Protocol
Date Submitted by the Author:	22-Aug-2019
Complete List of Authors:	Ribbink, Marthe; Amsterdam University Medical Centres, Department of Internal Medicine, Section of Geriatric Medicine<br>Macneil-Vroomen , Janet; Amsterdam University Medical Centres, Department of Internal Medicine, Section of Geriatric Medicine ; Yale School of Medicine, Department of Internal Medicine, Section of Geriatrics<br>van Seben, Rosanne; Amsterdam University Medical Centres, Department of Internal Medicine, Section of Geriatric Medicine<br>Oudejans , Irène; Amsterdam University Medical Centres, Department of Internal Medicine, Section of Geriatric Medicine<br>Buurman, Bianca; Amsterdam University Medical Centres, Department of Internal Medicine, Section of Geriatric Medicine ; Amsterdam University of Applied Sciences, ACHIEVE- Centre of Applied Research, Faculty of Health
Keywords:	GERIATRIC MEDICINE, OLDER ADULTS, COMMUNITY HOSPITAL, INTERMEDIATE CARE FACILITIES, READMISSIONS, FUNCTIONAL DECLINE

SCHOLARONE™
Manuscripts

I, the Submitting Author has the right to grant and does grant on behalf of all authors of the Work (as defined in the below author licence), an exclusive licence and/or a non-exclusive licence for contributions from authors who are: i) UK Crown employees; ii) where BMJ has agreed a CC-BY licence shall apply, and/or iii) in accordance with the terms applicable for US Federal Government officers or employees acting as part of their official duties; on a worldwide, perpetual, irrevocable, royalty-free basis to BMJ Publishing Group Ltd ("BMJ") its licensees and where the relevant Journal is co-owned by BMJ to the co-owners of the Journal, to publish the Work in this journal and any other BMJ products and to exploit all rights, as set out in our licence.

The Submitting Author accepts and understands that any supply made under these terms is made by BMJ to the Submitting Author unless you are acting as an employee on behalf of your employer or a postgraduate student of an affiliated institution which is paying any applicable article publishing charge ("APC") for Open Access articles. Where the Submitting Author wishes to make the Work available on an Open Access basis (and intends to pay the relevant APC), the terms of reuse of such Open Access shall be governed by a Creative Commons licence – details of these licences and which Creative Commons licence will apply to this Work are set out in our licence referred to above.

Other than as permitted in any relevant BMJ Author's Self Archiving Policies, I confirm this Work has not been accepted for publication elsewhere, is not being considered for publication elsewhere and does not duplicate material already published. I confirm all authors consent to publication of this Work and authorise the granting of this licence.

For peer review only

Evaluating an acute geriatric community hospital for older adults in a prospective cohort study compared with two historical control groups: a study protocol

Marthe E. Ribbink MD[1], Janet L. MacNeil Vroomen PhD[1,2], Rosanne van Seben PhD[1], Irène Oudejans MD, PhD[1], Bianca M. Buurman RN, PhD[1,3]

[1] Amsterdam University Medical Centre, University of Amsterdam, Department of Internal Medicine, Section of Geriatric Medicine, Amsterdam Public Health Research Institute, Meibergdreef 9, Amsterdam, The Netherlands

[2] Section of Geriatrics, Department of Internal Medicine, School of Medicine, Yale School of Medicine, The United States

[3] ACHIEVE – Centre of Applied Research, Faculty of Health, Amsterdam University of Applied Sciences, Amsterdam, The Netherlands

Word count, references and tables

Abstract: 271 words (max 300)

Total word count: 3998 words (max 4000)

Number of references: 65 (max 100)

Number of tables: 2 (box 1 and table 1)

Email addresses authors

M.E. Ribbink, m.e.ribbink@amsterdamumc.nl

J.L MacNeil Vroomen j.l.macneil-vroomen@amsterdamumc.nl

R. van Seben rosanne.vanseben@ecorys.com

I. Oudejans i.oudejans@amc.uva.nl

B.M. Buurman b.m.vanes@amsterdamumc.nl

Corresponding author

M.E. Ribbink

Amsterdam UMC, University of Amsterdam, Department of Internal Medicine, Section of Geriatric Medicine, D3-335

PO Box 22600, 1100 DD Amsterdam, The Netherlands

Tel: 0031-20 5661647, Email: m.e.ribbink@amsterdamumc.nl

For peer review only

Keywords: geriatric medicine, older adults**,** community hospital, intermediate care facilities, readmissions, functional decline.

Abstract

Introduction: Hospital admission is associated with unwanted outcomes like readmission, institutionalization, functional decline, and mortality in older adults with multiple chronic conditions. Providing acute care in the community and integrating effective components of care models might lead to a reduction of negative outcomes. Recently, the first geriatrician-led care Acute Geriatric Community Hospital (AGCH) was introduced in the Netherlands. Care at the AGCH is focused on: treatment of acute disease, comprehensive geriatric assessment, setting patient-led goals, early rehabilitation and stream-lined transitions of care.

Methods and analysis: This prospective cohort study compared with two historical control groups will investigate the effectiveness of care delivery at the AGCH on patient outcomes, by comparing AGCH patients to hospital patients. Propensity score matching will correct for potential population differences. The primary outcome is the three month unplanned readmission rate. Secondary outcomes include: functional decline, institutionalization, healthcare utilization, occurrence of delirium or a fall, health-related quality of life, mortality and patient satisfaction. Measurements will be conducted at admission, discharge and one, three and six months after discharge. Furthermore, an economic evaluation and qualitative process evaluation to assess facilitators and barriers for implementation is planned.

Ethics and dissemination: The study will be conducted according to the Declaration of Helsinki. The Medical Ethics Research Committee (METC) confirmed that the Medical Research Involving Human Subjects Act did not apply to this research project and official approval was not required. The findings of this study will be disseminated through academic and public lectures, scientific conferences and in peer-reviewed journals. Furthermore, the findings of this study will aid in the implementation and financing of this concept (inter)nationally.

Trial Registration Number NL7896; pre-results

For peer review only

Strengths and limitations of this study:

- The strengths of this proposed study include: a mixed-methods evaluation of hospital, patient-reported and economic outcomes; aiming to evaluate this complex intervention versus care as usual.
- Further strengths involve including patients and informal caregivers in the design, financing and implementation of the Acute Geriatric Community Hospital.
- Limitations associated with the design include: selection of appropriate controls from the two historic cohorts, potential follow-up response rates to the questionnaires (conducted by telephone), organizational challenges on chart review and collecting data on readmissions.

For peer review only

Introduction

Background

Throughout the western world, there is an increase of older adults requiring acute care. Inpatient services are mostly consumed by those over the age of 65.[1,2] The Netherlands, like many other countries, recently (2015) implemented stay-at-home policies leading to an increase of frail older persons living longer in the community.[3] These reforms juxtaposed with an increased aging population, contribute to increased acute care utilization.[4] There has been a 19% increase in emergency department visits by Dutch older adults based on data from 2015 versus 2017.[5,6]

Many older adults come to the hospital with complex and atypical health problems.[5,7] When older persons are subsequently hospitalized, health outcomes are known to be poor,[8] particularly in patients with geriatric syndromes such as cognitive impairment or mobility impairment.[9,10] For example, previous research showed that 30% of older persons gained new disabilities and 20% were readmitted within 30 days postdischarge.[11,12] Hospitalization itself may contribute to these poor outcomes as hospital older adult inpatients often have reduced mobility while bedbound for approximately 20 hours daily.[13,14] Low physical activity, in combination with poor nourishment and increased caloric demand due to acute illness can lead to loss of muscle mass and may contribute to the development of new disabilities, particularly in frail patients.[15,16] Together with noise in a hospital environment and different personnel rotating through patient rooms, this contributes to sensory overstimulation and sleep deprivation, which may lead to confusion and the occurrence of delirium.[17,18,19] Not only is the patient affected during hospitalization, informal caregivers also find hospital admissions stressful.[20] Furthermore, previous research showed that lack of discharge planning in the hospital can result in the care needs of patients being unmet.[21] Hospital care as usual compared to discharge planning and follow-up showed an increased on early readmissions.[22] Readmissions can further effect patients recovery and increase healthcare costs.[23]

For peer review only

The complex medical needs of older persons, combined with their more dependent social situation requires care delivery that offers guidance and support of realistic health and life goals.[24] Perhaps a 'gap' exists between what care can be provided in hospital, that is specialist care, with a focus on medical treatment and diagnostics, versus what can be provided in the community, that is primary care focused on rehabilitation, nursing care and wellbeing.

Several alternative strategies to hospital admission and (nurse-led) intermediate care have been developed in the past as a substitute to conventional hospitalization.[25] Examples include (nurse-led) intermediate care and subacute geriatric care units, low-tech but with geriatric expertise.[26,27] In general, these types of care have comparable outcomes to hospital care as usual. Moreover, nurse-led care in the US, observation units and hospital at home care all showed a cost reduction compared to care as usual.[25,26] Until recently, the Netherlands however, had limited alternatives to hospitalization for older persons who required acute care. Therefore, our research group sought to create an acute care alternative and opened the Acute Geriatric Community Care Hospital (AGCH) in July 2018, partnering with an academic hospital, an insurance company and a home care agency. This acute geriatric care unit, which is based within an intermediate care facility, provides an alternative to conventional hospitalization and delivers acute care closer to home.

The AGCH delivers hospital care that is focused on early mobilization and rehabilitation. Older persons with common medical problems (such as urinary tract infections, pneumonia or heart failure) and geriatric syndromes requiring hospital admission can be admitted to the AGCH. The AGCH provides a form of *intermediate* care between primary and secondary care. In the Netherlands, primary care includes general practice, community nursing and (temporary) admission to nursing home. Secondary care includes medical specialist care and hospital admission. Care is supervised by a geriatrician and provided by nurses trained in geriatric care who have experience as either a hospital or community nurse. The rooms are designed to accommodate respite for the informal caregivers.

For peer review only

Our hypothesis is that with the provision of integrated medical and nursing care close to home, the AGCH is better suited to the needs of older adults with multiple chronic conditions and will lead to better patient health outcomes and reduced post-acute care costs. Therefore, this study is designed to compare care provided for older patients in the AGCH versus care provided in a hospital setting. Specifically we aim to:

- Evaluate the 90-day readmission rate of patients acutely admitted to AGCH compared to patients admitted a traditional hospital (usual care). Secondary outcomes include: functional decline, institutionalization, healthcare utilization, the occurrence of geriatric syndromes such as delirium, health-related quality of life, mortality, and patient satisfaction;
- Assess the cost-effectiveness of the AGCH versus usual care by performing an economic evaluation from a health care provider and societal perspective;
- Conduct a process evaluation using interviews with key stakeholders to identify facilitators and barriers to the implementation of the AGCH.

Methods

Setting

The Acute Geriatric Community Hospital opened in July 2018. It serves both the south-eastern part of Amsterdam and its surrounding areas (an area with approximately 147 500 inhabitants).[28] The AGCH is a 20 -bed facility within an skilled nursing facility. The hospital has 24-hour geriatric and nursing assistance, physiotherapy and routine laboratory testing during the workweek and simple x-ray available once a week. The population that is eligible for admission to the AGCH includes patients with 1) acute medical problems requiring hospitalization (e.g. pneumonia, exacerbation of heart failure or an urinary tract infection) 2) geriatric conditions (e.g. delirium, cognitive impairment, falls, functional impairment), who are 3) hemodynamically stable and 4) not in need of complex diagnostic testing. In general, patients will not be admitted if they: 1) require care that can only be provided at

For peer review only

an intensive care unit 2) require surgery 3) require urgent treatments or diagnostic tests that can only be provided in-hospital (e.g. endoscopy, interventional radiology) 4) do not need hospital care, but require transfer to a skilled nursing facility and 5) live in another region of the Netherlands. Patients are directly admitted to the AGCH from the emergency department (ED) of the Amsterdam UMC- location Academic Medical Centre (AMC) in Amsterdam which is a 1000-bed academic hospital with approximately 30,000 ED visits yearly. After the on-call geriatrician has assessed that patient is eligible for AGCH admission and the patient or representative has agreed to admission, the patient is transferred to the AGCH by ambulance. Patients are admitted between 8.00 am and 11.00 pm, 7 days a week. At admission, a Comprehensive Geriatric Assessment (CGA) is conducted.[29] The CGA gives an overview of all medical, functional, psychological and social problems that are discussed during multidisciplinary team meetings and are used to formulate a care plan for each patient.

Study design

This study is a prospective, observational, cohort study with two historical control groups to evaluate the clinical and economic effects of the AGCH. The STROBE statement was used in preparing the study protocol.[30] Participants will be compared to hospital controls. The participants are recruited into the study and are assessed at admission, discharge, one month, three and six months after discharge. The recruitment phase of this study started in February 2019. We plan to recruit for 18 to 24 months. The first three months of data collection will consist of a piloting phase to assess the feasibility of data-collection and follow-up. Moreover, we will assess the level of implementation of the AGCH care program alongside our investigation (see box 1). In addition, a qualitative process evaluation for the study on facilitators and barriers of the implementation of the AGCH will be conducted.

For peer review only

Participants

Patients admitted to the AGCH are eligible for inclusion to the study. However, patients are excluded from the study if: 1) the attending physician judges that the patient is too ill to participate e.g. is terminally ill 2) the patient or legal representative does not consent to participate. 3) the patient or legal representative does not speak or understand Dutch or English. In the case of cognitively impaired or delirious patients, patients can only be included if a legal representative consents to participation and participates on their behalf. Cognitive functioning or the presence of delirium is assessed by the attending physician and confirmed by the researcher by conducting a Mini Mental State Exam (MMSE). A MMSE score of 15 or less Indicates severe cognitive impairment, in which the approval of a legal representative will be sought.[31]

Historical control groups

The first control group from the Transitional Care Bridge Study consists of 674 patients that were recruited between September 2010 and March 2014 originating from the greater Amsterdam area in the Netherlands.[32] Participants were patients of 65 years and older admitted for at least 48 hours to an internal medicine ward. Proxy consent was provided for participants suffering from severe cognitive impairment: Mini Mental State Exam[31] (MMSE) ≤15. They participated in a negative randomized controlled trial that assessed the effectiveness of a nurse-led transitional care program in preventing functional decline.[32]

The second control group (Hospital-ADL study[10]) consists of 401 patients that were recruited between October 2015 and June 2017 also originating from the greater Amsterdam area. These participants were enrolled in a prospective cohort studying the trajectory of functional decline in older hospitalized adults. Participants were aged 70 years and older, hospitalized for at least 48 hours. Patients suffering from severe cognitive impairment (MMSE ≤15) and delirium were excluded from participation.

For peer review only

Patient and public involvement

Older persons living in the Amsterdam area were involved in the design of the AGCH concept. There was no patient involvement in the design of this study.

Outcomes

The primary outcome measure is the 3-month unplanned readmission rate to the AGCH or hospital. Secondary outcomes measured at 1,3 and 6 months will include:

1) ADL-functioning as defined by the KATZ-ADL scale.[33]

2) Healthcare utilization, including institutionalization in a long-term care facility.

3) Occurrence of delirium and/or falls.

4) Health-related quality of life (HRQoL).[34]

5) All-cause mortality.

6) Satisfaction of patients and primary care givers with the care provided.

Data collection

Eligible patients and/or legal representatives will be contacted and informed about the study procedures after which written informed consent is obtained. Inclusion and interviewing of patients is conducted by an onsite researcher. Routine data on functioning and risk assessments are collected by a trained registered nurse and physiotherapist as part of the CGA for each patient.[35] Table 1 gives an overview of measurement of primary and secondary outcomes over time. Measurements during admission are at H1 which is within 48 hours after admission and H2 which is within 48 hours before discharge. Follow-up is completed by telephone at 1,3 and 6 months after discharge (P1, P3 and P6).

Data collection includes:

1. Medical and demographical data

For peer review only

Sociodemographic data. These will include age, gender, highest level of education, ethnicity, marital status and living arrangement.

Time spent at the ED, admission diagnosis, date and time of admission.

Chronic conditions. The number and severity of chronic conditions will be assessed using the Charlson Comorbidity Index.[36] This index is commonly used to indicate the risk of mortality; each condition is scored 1, 2, 3 or 6 points, with a higher total number of points indicating a greater risk at death.

Polypharmacy. Polypharmacy will be assessed by counting the number of individual drugs that are chronically prescribed to a participant, in which a number of 5 or more drugs is considered polypharmacy.

Mortality. This will assessed during follow-up, where possible by reporting from patients electronic files, otherwise from registries of the general practitioner.

2. Cognitive functioning.

Cognitive impairment. This will be is assessed by reviewing the score of the Mini Mental State Exam (MMSE) that is performed within 48 hours of admission. The MMSE includes 23 items (total score 0-30) that screen for cognitive impairment. A score of 23 or less is defined as possible cognitive impairment.[31]

Delirium. The Confusion Assessment Method (CAM), the short 4 item version, is used to assess the presence and duration of delirium.[37] The CAM is widely used by physician and nurse practitioners to diagnose delirium (sensitivity of 53-90% and specificity of 84-100%).[38] It consists of four items: 1) presence of acute onset and fluctuation 2) inattention 3) disorganized thinking and 4) altered level of consciousness.[37] The CAM is filled out within 24 hours of admission. Moreover, the risk on developing delirium is assessed using the Dutch VMS criteria for risk on delirium.[39] Nurse practitioners will score the CAM daily from day 1 till day 8 of admission, if there are signs of possible

For peer review only

delirium at day 8, these measurements are continued until discharge. In addition, during the first three days of admission the Delirium Observation Screening Scale (DOSS) is scored during each nursing shift and is continued when there is a clinical suspicion of delirium.[40]

3. Psychosocial functioning and quality of life

Apathy. We use three items of the *Geriatric Depression Scale* (GDS-15) to assess apathy (sensitivity of 69% and specificity of 85 %). These items include the following questions: 1) 'Do you prefer to stay at home, rather than going out and doing new things' 2) 'Have you dropped many of your activities and interests?' And 3) 'Do you feel full of energy'. A score of >2 points is classified as 'apathy present'. [41]

Social network and informal care. Participants are asked if they receive informal care, how many hours a week, what type of care (housekeeping and/or personal care) and from which persons (partners, children, other family members or neighbours/volunteers).

Health-Related Quality of Life. This will be measured by determining Health-Related Quality of Life using the EuroQoL-5D (EQ-5D). The EQ-5D is a broadly used and validated instrument for measuring generic health-related quality of life.[34] It consists of 5 dimensions: 1) mobility 2) self-care 3) usual activities 4) pain/discomfort 5) anxiety/depression. We will use the EQ-5D-3L which has three options: no problems, some problems or severe problems. In addition, the following questions will be used to assess self-reported quality of life: 1) How would you rate your quality of life in general ? (excellent, very good, good, moderate, bad) 2) How would you rate your quality of life in general at this time compared to 6 months ago? (much better, somewhat better, more or less the same, somewhat worse, much worse) 3) How would you grade your life at this moment, with a range between 0 and a 100? [42]

4. Physical functioning

For peer review only

Risk of functional decline. Patients are assessed for risk of functional decline using the Identification of Seniors at Risk- Hospitalized patients (ISAR-HP), scores of two and up indicate at an increased risk for functional decline.[43]

Functioning level. The 15-item modified KATZ score is used to measure Activities of Daily Living (ADL)-functioning. This included assessment of performance in basic ADL- KATZ-6), as in instrumental ADL (KATZ-9).[44,45] We measure KATZ-score both currently (at admission), as two weeks before admission, reflecting pre-morbid level of functioning. (I)ADL-functional is also included in follow-up measurements.

(Im)mobility. We will assess mobility by reviewing three questions that are in the admission assessment regarding: 1) use of a walking aid (from KATZ-15), and from the CGA: 2) being able to walk outside of the house for five minutes (two weeks before and currently) and 3) performing, and frequency of, physical activity.[46]

Handgrip strength. Physiotherapists measure muscle weakness in all admitted patients using maximum handgrip strength (JAMAR). [47]

Gait speed. Gait speed is measured as part of the Short Physical Performance Battery (SPBB) that is part of the physiotherapist' admission assessment.[48]

Falls. Fall history is assessed by asking the number of falls in the past six months, if yes, how many times did you fall?[39] During the discharge assessment the occurrence of falls in the AGCH and the consequences of falls (indication for prolonged stay, diagnostics or injury) are recorded.

Fear of falling. We will use a Numeric Rating Scale (NRS, score 0-10) to assess fear of falling, 0 indicates no fear of falling, and 10 the greatest fear of falling possible.[49]

Pain. Widely used in clinical practice the standard for pain assessment is the Numeric Rating Scale, ranging from 0 to 10, in which a score of 0 represents no pain and 10 represents the worst possible pain.[50]

For peer review only

Fatigue. A Numeric Rating Scale from 0-10 is used, 0 indicating no fatigue and 10 indicating the greatest fatigue ever felt by the participant.[51]

Sleep. Participants are asked if there have been difficulties with sleeping in the past month and whether participants have used sleep medication.

Nutrition. We will use the Short Nutritional Assessment Questionnaire (SNAQ) for identifying malnourished patients. The SNAQ consists of three questions on weight loss, appetite and drink/tube nutrition, resulting in a score ranging from 0 to 5. 0 and 1 are defined as 'no malnutrition' 2 as 'moderate malnutrition' and 3 or more as 'severe malnutrition'.[52]

5. Healthcare utilization and satisfaction with care

Medical care during admission and process of discharge. The following are collected from patient electronic health records: the diagnostics performed in the AGCH, revisits to the hospital, admission to the hospital, length of stay of the AGCH, discharge destination and time needed to send medical handovers to the general practitioner.

Hospital readmission. This outcome will be assessed during follow-up. Follow-up will consist of three telephone interviews at 1, 3 and 6 months after discharge. Readmission will be both assessed by interview as by checking care data from an aggregated database of expense claims of various healthcare insurers. Data that will be collected are: number of readmissions, total days of readmission, reasons for readmission and whether the readmission was planned or unplanned.

Emergency department (ED) visits. ED visits will be assessed during follow-up and checked in insurance data, we will record the number of separate ED visits.

Outpatient hospital visits. We will ask patients if there have been any outpatient visits in the past month(s), and if so how many.

For peer review only

Consultations by general practitioner. We will ask patients if there have been any consultations by the general practitioner (both during the day as during out-of-office hours) visits in the past month(s), and if so how many.

Consultations by physiotherapist or dietician. We will ask patients if there have been any consultations by a physiotherapist of dietician in the past month(s), and if so how many.

Home care. This includes questions on frequency of home care, including housekeeping, personal care and nursing care. We will also include days of day care and hours of informal care provided by family members or friends.

Temporary admission to a nursing home. Days of (temporary) admission to a skilled nursing facility or rehabilitation facility.

Permanent Institutionalization. This concerns long-term admission to a skilled nursing facility and date of admission to this facility.

Patient satisfaction with care. Patients or informal caregivers are asked to fill out an 8-question questionnaire regarding their satisfaction with the care that they received. Questions are answered on a 5- level Likert scale.[53]

Sample Size calculation

The dataset of the transitional care bridge includes data of 674 patients of conventionally hospitalized; approximately 26% experienced a readmission at 90 days.[32] Assuming that 19% of patients admitted to the AGCH will experience a 90 day readmission, data from 523 patients of AGCH will give us 80% power to detect an absolute difference of 7% in readmission rate (which is a 27% reduction in relative risk) using a two-sided Fisher's Exact Test with an alpha of 0.05. As we expect 10% loss to follow-up, we will aim to include a total of 576 (= 523*1.1) patients from the AGCH.

For peer review only - http://bmjopen.bmj.com/site/about/guidelines.xhtml

Planned statistical analysis

The participant flow diagram according to the STROBE guidelines will show a summary of admissions and study recruitment at the AGCH and will provide study discontinuation rates at 1, 3 and 6 months follow-up.[30] We will describe demographic, clinical and prognostic characteristics of the study participants at baseline. The number of participants with missing data will be collected and described alongside our variables to check for the pattern of missingness. Inversely-weighted propensity scores will be used to control for any imbalances between the treatment groups.[54] Propensity scores will be calculated using generalized booted methods. Balance and overlap of propensity scores distribution will be assessed. Propensity score weights for the estimation of the average treatment effect will be created using all covariates where groups differed on baseline or that were associated with 90-day readmission rate. As this is a repeated measures design, we will assume equal weighting for all measurements.[55]

All hypotheses will be tested using two-tailed- significance level of 0.05. Descriptive analyses will be performed to examine participant's characteristics. Differences in changes over time in outcomes will be compared between groups using multilevel models. All models will include a main effect of treatment group, a linear term for time and an interaction between time and treatment group. Models will be checked with residual and appropriate goodness-of-fit statistics.

Economic evaluation

A healthcare and societal perspective is planned for this economic evaluation.[56] The evaluation from the healthcare perspective will only include direct medical costs accrued in the six months after the admission to the AGCH. Propensity scores will also be used in the economic evaluation. Missing data will be imputed using multiple imputation chained equations if necessary, for cost and effect data. We plan to use generalized linear regression models with a gamma distribution and an identity link to account for the right skew of cost data. A generalized linear regression model will be used to

For peer review only

estimate the incremental effect in quality adjusted life years (QALYs) adjusted for baseline utility estimates with a Gaussian distribution and identify link.[57] Incremental cost-effectiveness ratios will be calculated using the pooled cost and effect estimates. Bootstrapped cost-effect pairs will be plotted on a cost-effectiveness plane and used to estimate cost-effectiveness acceptability curves.[58]

Process evaluation; adherence, barriers and facilitators to implementation.

We plan to use a qualitative study design to describe barriers and facilitators to implementation of the AGCH. This will include semi-structured interviews with various stakeholders, emergency department staff, geriatricians, nurses, physiotherapists, discharge nurses, home care, hospital administrators, and insurance companies. We will assess barriers and facilitators to implementation at different levels: micro (healthcare professionals), meso (care organizations) and macro level (legal and financial framework). Interviews will be typed verbatim and analysed by two researchers independently, using thematic analysis.[59] The findings will be summarized in matrices with facilitators and barriers at different levels (micro, meso, macro) to develop a guideline for implementation of the AGCH elsewhere.[60]

Discussion

The complex acute medical needs of older patients require the delivery of specialized geriatric care, however the traditional hospital environment may not support recovery and maintaining independence. The AGCH aims to deliver care that focusses both on medical treatment, early rehabilitation and proper transitions of care for older adults with multiple chronic conditions.[29,61] The AGCH is unique in the Netherlands in its aim to combine multiple evidenced-based components of care for frail older persons in an alternative location for hospital care. The proposed research will provide insight into the clinical and economic effectiveness of care delivered at the AGCH, compared to hospital care. Limitations to the design are that it is non-randomized study and that historic cohorts are used as control groups. Strengths are that patients and informal caregivers were

For peer review only

involved in the design of the concept of the AGCH. Moreover, a process evaluation will address the barriers and facilitators to implementation of a community hospital such as the AGCH in the existing health care system of the Netherlands. This research will provide valuable insights into the implementation of this concept of care in other regions of the Netherlands and abroad.

Ethics and Dissemination

This trial will be carried out in accordance with the declaration of Helsinki and current ethical requirements. The Medical Ethics Research Committee (METC) confirmed that the Medical Research Involving Human Subjects Act did not apply to this research project and official approval was not required. The outcomes of this trial will be reported according to STROBE guidelines for cohort studies.[30] This study will evaluate both the effectiveness of this type of care delivery as of the costs that are involved, allowing for implementation elsewhere. The findings of this study will be published in peer-reviewed journals.

Acknowledgements
The authors thank all (care) professionals from the Amsterdam University Medical Centres, Cordaan and Zilveren Kruis who have worked on the development of the AGCH. Thank you for your time advise and cooperation.

Author contributions
MER, JMV and RS contributed to the design of the protocol and drafting of the manuscript. IO and BMB made substantial contributions to the design and clinical aspects of the of the protocol. BMB conceived the study and wrote funding applications. All authors critically revised the manuscript and approved the final version of this manuscript.

Funding statement
This research received no specific grant from any funding agency in the public, commercial or not-for-profit sectors. The care provided at the AGCH (WijkKliniek) is provided in a partnership between Cordaan, a community and home-care organization and the Amsterdam University Medical Centres, location Academic Medical Centre. The AGCH (WijkKliniek) is financially supported by Zilveren Kruis, a health insurance company.

Patient consent
Not required.

For peer review only

Competing interests statement- None Declared

Ethics approval –The Medical Ethics Research Committee (METC) of the Amsterdam University Medical Centres, location Amsterdam Medical Centre (AMC) confirmed that the Medical Research Involving Human Subjects Act did not apply to this research project and official approval was not required, January 2019. (ref W17_474 # 19.001)

References

1. McCabe JJ, Kennelly SP. Acute care of older patients in the emergency department: strategies to improve patient outcomes. *Open Access Emerg Med.* 2015;7:45-54.
2. Centraal Bureau voor de Statistiek. Ziekenhuisopnamen; kerncijfers; geslacht, leeftijd en regio. http://statline.cbs.nl/StatWeb/publication/?DM=SLNL&PA=71857ned. Published 2014. Accessed.
3. Government of the Netherlands Ministry of Health Welfare and Sport- Living Independently for Longer https://www.government.nl/topics/care-and-support-at-home/living-independently-for-longer. Published 2019. Accessed.
4. Jayaprakash N, O'Sullivan R, Bey T, Ahmed SS, Lotfipour S. Crowding and delivery of healthcare in emergency departments: the European perspective. *West J Emerg Med.* 2009;10(4):233-239.
5. de Kousemaeker G, Vandermeulen L. *Ontwikkelingen Langdurige Zorg* Fluent Healthcare Amsterdam 2017.
6. DeFrances CJ, Lucas CA, Buie VC, Golosinskiy A. 2006 National Hospital Discharge Survey. *National health statistics reports.* 2008(5):1-20.
7. Boyd CM, Xue QL, Guralnik JM, Fried LP. Hospitalization and development of dependence in activities of daily living in a cohort of disabled older women: the Women's Health and Aging Study I. *The journals of gerontology Series A, Biological sciences and medical sciences.* 2005;60(7):888-893.
8. Buurman BM, Han L, Murphy TE, et al. Trajectories of Disability Among Older Persons Before and After a Hospitalization Leading to a Skilled Nursing Facility Admission. *Journal of the American Medical Directors Association.* 2016;17(3):225-231.
9. Fogg C, Griffiths P, Meredith P, Bridges J. Hospital outcomes of older people with cognitive impairment: An integrative review. *International journal of geriatric psychiatry.* 2018.
10. van Seben R, Reichardt LA, Aarden J, et al. Geriatric syndromes from admission to 3 months post-discharge and their association with recovery. *Innovation in Aging.* 2017;1(suppl_1):902-903.
11. Boyd CM, Landefeld CS, Counsell SR, et al. Recovery of activities of daily living in older adults after hospitalization for acute medical illness. *Journal of the American Geriatrics Society.* 2008;56(12):2171-2179.
12. Kansagara D, Englander H, Salanitro A, et al. Risk prediction models for hospital readmission: a systematic review. *Jama.* 2011;306(15):1688-1698.
13. Brown CJ, Redden DT, Flood KL, Allman RM. The underrecognized epidemic of low mobility during hospitalization of older adults. *Journal of the American Geriatrics Society.* 2009;57(9):1660-1665.
14. Agmon M, Zisberg A, Gil E, Rand D, Gur-Yaish N, Azriel M. Association Between 900 Steps a Day and Functional Decline in Older Hospitalized Patients. *JAMA internal medicine.* 2017;177(2):272-274.

For peer review only

15. Kortebein P, Symons TB, Ferrando A, et al. Functional impact of 10 days of bed rest in healthy older adults. *The journals of gerontology Series A, Biological sciences and medical sciences.* 2008;63(10):1076-1081.
16. Zisberg A, Shadmi E, Gur-Yaish N, Tonkikh O, Sinoff G. Hospital-associated functional decline: the role of hospitalization processes beyond individual risk factors. *Journal of the American Geriatrics Society.* 2015;63(1):55-62.
17. Missildine K, Bergstrom N, Meininger J, Richards K, Foreman MD. Sleep in hospitalized elders: a pilot study. *Geriatric nursing (New York, NY).* 2010;31(4):263-271.
18. Missildine K, Bergstrom N, Meininger J, Richards K, Foreman MD. Case studies: is the sleep of hospitalized elders related to delirium? *Medsurg Nurs.* 2010;19(1):39-46.
19. Dasgupta M, Brymer C. Poor functional recovery after delirium is associated with other geriatric syndromes and additional illnesses. *International psychogeriatrics.* 2015;27(5):793-802.
20. Rodakowski J, Rocco PB, Ortiz M, et al. Caregiver Integration During Discharge Planning for Older Adults to Reduce Resource Use: A Meta-analysis. *Journal of the American Geriatrics Society.* 2017.
21. Holland DE, Mistiaen P, Bowles KH. Problems and unmet needs of patients discharged "home to self-care". *Professional case management.* 2011;16(5):240-250; quiz 251-242.
22. Naylor MD, Brooten D, Campbell R, et al. Comprehensive discharge planning and home follow-up of hospitalized elders: a randomized clinical trial. *Jama.* 1999;281(7):613-620.
23. Mechanic R. Post-acute care--the next frontier for controlling Medicare spending. *The New England journal of medicine.* 2014;370(8):692-694.
24. Westphal EC, Alkema G, Seidel R, Chernof B. How to Get Better Care with Lower Costs? See the Person, Not the Patient. *Journal of the American Geriatrics Society.* 2016;64(1):19-21.
25. Conley J, O'Brien CW, Leff BA, Bolen S, Zulman D. Alternative Strategies to Inpatient Hospitalization for Acute Medical Conditions: A Systematic Review. *JAMA internal medicine.* 2016;176(11):1693-1702.
26. Griffiths PD, Edwards MH, Forbes A, Harris RL, Ritchie G. Effectiveness of intermediate care in nursing-led in-patient units. *The Cochrane database of systematic reviews.* 2007(2):CD002214.
27. Inzitari M, Gual N, Roig T, et al. Geriatric Screening Tools to Select Older Adults Susceptible for Direct Transfer From the Emergency Department to Subacute Intermediate-Care Hospitalization. *Journal of the American Medical Directors Association.* 2015;16(10):837-841.
28. Stadindex. Stadindex- Noord- Holland https://www.stadindex.nl/noord-holland. Published 2019. Accessed 11th of June 2019
29. Stuck AE. [Multidimensional geriatric assessment in the acute hospital and ambulatory practice]. *Schweizerische medizinische Wochenschrift.* 1997;127(43):1781-1788.
30. von Elm E, Altman DG, Egger M, et al. The Strengthening the Reporting of Observational Studies in Epidemiology (STROBE) statement: guidelines for reporting observational studies. *Journal of clinical epidemiology.* 2008;61(4):344-349.
31. Folstein MF, Folstein SE, McHugh PR. "Mini-mental state". A practical method for grading the cognitive state of patients for the clinician. *Journal of psychiatric research.* 1975;12(3):189-198.

32. Buurman BM, Parlevliet JL, Allore HG, et al. Comprehensive Geriatric Assessment and Transitional Care in Acutely Hospitalized Patients: The Transitional Care Bridge Randomized Clinical Trial. *JAMA internal medicine.* 2016;176(3):302-309.

33. Katz S. Assessing self-maintenance: activities of daily living, mobility, and instrumental activities of daily living. *Journal of the American Geriatrics Society.* 1983;31(12):721-727.

34. EuroQol--a new facility for the measurement of health-related quality of life. *Health policy (Amsterdam, Netherlands).* 1990;16(3):199-208.

35. Ellis G, Whitehead MA, Robinson D, O'Neill D, Langhorne P. Comprehensive geriatric assessment for older adults admitted to hospital: meta-analysis of randomised controlled trials. *BMJ (Clinical research ed).* 2011;343:d6553.

36. Charlson ME, Pompei P, Ales KL, MacKenzie CR. A new method of classifying prognostic comorbidity in longitudinal studies: development and validation. *Journal of chronic diseases.* 1987;40(5):373-383.

37. Inouye SK, van Dyck CH, Alessi CA, Balkin S, Siegal AP, Horwitz RI. Clarifying confusion: the confusion assessment method. A new method for detection of delirium. *Annals of internal medicine.* 1990;113(12):941-948.

38. NVKG-Dutch-Society-for-Clinical-Geriatrics. Meetinstrumenten voor diagnose delier. https://richtlijnendatabase.nl/richtlijn/delier_bij_volwassenen/meetinstrumenten_voor_diagnose_delier.html#onderbouwing. Published 2013. Accessed 8th of January 2019.

39. Heim N, van Fenema EM, Weverling-Rijnsburger AW, et al. Optimal screening for increased risk for adverse outcomes in hospitalised older adults. *Age and ageing.* 2015;44(2):239-244.

40. Schuurmans MJ, Shortridge-Baggett LM, Duursma SA. The Delirium Observation Screening Scale: a screening instrument for delirium. *Res Theory Nurs Pract.* 2003;17(1):31-50.

41. van der Mast RC, Vinkers DJ, Stek ML, et al. Vascular disease and apathy in old age. The Leiden 85-Plus Study. *International journal of geriatric psychiatry.* 2008;23(3):266-271.

42. TOPICS. Minimale Data Set Zorgvrager Basismeting. https://topics-mds.eu/survey-instrument/questionnaires/nederlands/. Accessed 8th of January 2019.

43. Hoogerduijn JG, Schuurmans MJ, Duijnstee MS, de Rooij SE, Grypdonck MF. A systematic review of predictors and screening instruments to identify older hospitalized patients at risk for functional decline. *Journal of clinical nursing.* 2007;16(1):46-57.

44. Katz S, Downs TD, Cash HR, Grotz RC. Progress in development of the index of ADL. *The Gerontologist.* 1970;10(1):20-30.

45. Laan W, Zuithoff NP, Drubbel I, et al. Validity and reliability of the Katz-15 scale to measure unfavorable health outcomes in community-dwelling older people. *The journal of nutrition, health & aging.* 2014;18(9):848-854.

46. Stichting-effectieve-ouderenzorg. Comprehensive Geriatric Assesment TZB https://effectieveouderenzorg.nl/zorgmodellen/comprehensive-geriatric-assessment/. Published 2013. Accessed 8th of January 2019.

47. Roberts HC, Denison HJ, Martin HJ, et al. A review of the measurement of grip strength in clinical and epidemiological studies: towards a standardised approach. *Age and ageing.* 2011;40(4):423-429.

48. Guralnik JM, Simonsick EM, Ferrucci L, et al. A short physical performance battery assessing lower extremity function: association with self-reported disability and prediction of mortality and nursing home admission. *Journal of gerontology.* 1994;49(2):M85-94.

For peer review only

49. Reichardt LA, Aarden JJ, van Seben R, et al. Unravelling the potential mechanisms behind hospitalization-associated disability in older patients; the Hospital-Associated Disability and impact on daily Life (Hospital-ADL) cohort study protocol. *BMC geriatrics.* 2016;16(1):59.
50. McCaffery M, Beebe A. Pain: clinical manual for nursing practice. *CV Mosby: St Louis.* 1989.
51. Hwang SS, Chang VT, Cogswell J, Kasimis BS. Clinical relevance of fatigue levels in cancer patients at a Veterans Administration Medical Center. *Cancer.* 2002;94(9):2481-2489.
52. Kruizenga HM, Seidell JC, de Vet HC, Wierdsma NJ, van Bokhorst-de van der Schueren MA. Development and validation of a hospital screening tool for malnutrition: the short nutritional assessment questionnaire (SNAQ). *Clinical nutrition (Edinburgh, Scotland).* 2005;24(1):75-82.
53. Likert. A technique for the measurement of attitudes. In. Archives of Psychology, 140, 1–55.1932.
54. White IR, Royston P, Wood AM. Multiple imputation using chained equations: Issues and guidance for practice. *Stat Med.* 2011;30(4):377-399.
55. van Walraven C, Seth R, Austin PC, Laupacis A. Effect of discharge summary availability during post-discharge visits on hospital readmission. *Journal of general internal medicine.* 2002;17(3):186-192.
56. Rutten-van Molken M. *Van kosten tot effecten* Elsevier 2010.
57. Lamers LM, Stalmeier PF, McDonnell J, Krabbe PF, van Busschbach JJ. [Measuring the quality of life in economic evaluations: the Dutch EQ-5D tariff]. *Nederlands tijdschrift voor geneeskunde.* 2005;149(28):1574-1578.
58. Willan AR BA. *Statistical analysis of cost-effectiveness data.* John Wiley and Sons 2006.
59. Braun V, Clarke V. Using thematic analysis in psychology. *Qualitative research in psychology.* 2006;3(2):77-101.
60. Moore GF, Audrey S, Barker M, et al. Process evaluation of complex interventions: Medical Research Council guidance. *BMJ (Clinical research ed).* 2015;350:h1258.
61. van Seben R, Geerlings SE, Buurman BM. [10 tips for improving patient handovers from hospital to other health care settings]. *Nederlands tijdschrift voor geneeskunde.* 2017;161(0):D1031.
62. Verhaegh KJ, MacNeil-Vroomen JL, Eslami S, Geerlings SE, de Rooij SE, Buurman BM. Transitional care interventions prevent hospital readmissions for adults with chronic illnesses. *Health affairs (Project Hope).* 2014;33(9):1531-1539.
63. Dutch Health Care Inspectorate (IGZ). Continuïteit van zorg voor kwetsbare ouderen vanuit het ziekenhuis naar verpleeg- en verzorgingshuizen, thuiszorg en huisartsen niet gewaarborgd. . 2015.
64. van Seben R, Geerlings SE, Verhaegh KJ, Hilders CG, Buurman BM. Implementation of a Transfer Intervention Procedure (TIP) to improve handovers from hospital to home: interrupted time series analysis. *BMC health services research.* 2016;16:479.
65. Zorginsituut N. Veelgestelde vragen: polifarmacie https://www.gipdatabank.nl/veelgestelde-vragen/polyfarmacie. Published 2017. Accessed 8th of january 2019.

Box 1 - Components of Acute Geriatric Care (AGCH) Hospital intervention and goals

1) Patients receive a full CGA work-up and interdisciplinary assessment, including physiotherapy treatment plan.<br>2) There is special attention paid to discharge and follow-up planning; if needed, patients are seen post-discharge at the outpatient clinic or through a community nurse. Community nurses receive warm-handovers from AGCH staff.[62]<br>3) 50% of discharge letters are sent to the general practitioner within 24 hours after the patient is discharged.[63,64]

For peer review only

Table 1 Overview of the content and description of (outcome) measurements and timing of measurements at the Acute Community Care Hospital ('WijkKliniek')

	**Description and/or instrument**	**H1**	**H2**	**P1**	**P3**	**P6**
1. Medical and demographical data						
**Sociodemographic data**	Date of birth, age at admission, sex, level of education, living conditions, marital state	R				
**Data on admission**	Time spent at the ED, admission diagnosis, date and time of admission	R				
**Chronic conditions**	Charlson Comorbidity index[36]	R				
**Polypharmacy**	Number of drugs[65]	R				
**Mortality**	Date of death		R	R	R	R
2. Cognitive functioning						
**Cognitive impairment**	Mini Mental State Exam (MMSE)[31]	R				
**Delirium**	Safety management system patient screening (VMS)[39]<br>Confusion Assessment Method (CAM)[37]<br>Delirium Observation Scale (DOS)[40]	N/D	N/D			
3. Psychosocial functioning and quality of life						
**Apathy**	Geriatric Depression Scale (GDS-3)[41]	N	R	R	R	R
**Social network and informal care**	Presence and frequency of informal care	R		R	R	R
**Quality of life and health status**	EQ-5D-3L[34]	R		R	R	R
4. Physical functioning						
**Identifying at-risk-patients**	ISAR-HP- Identifying Seniors at Risk score[43]	N				
**Functional status**	Activities of daily Living (ADL) modified Katz-ADL score[33]	N				
**(Im)mobility**	Using walking aid, information in KATZ-15 questions on exercise	N				
**Handgrip strength**	Jamar[47]	P				
**Gait speed**	Short Physical Performance Battery SPPB[48]	P				
**Falling**	Fall history<br>Falls in the AGCH<br>Numeric Rating scale (NRS) fear of falling[49]	N<br><br>N	R<br><br>R	R<br><br>R	R<br><br>R	R<br><br>R
**Pain**	Numeric Rating Scale (NRS) pain [50]	N	R	R	R	R
**Fatigue**	Numeric Rating Scale (NRS) fatigue [51]	N	R	R	R	R
**Nutrition**	Short Nutritional Assessment Questionnaire (SNAQ- Score)[52]	N				
5. Healthcare utilization and satisfaction with care						
**Medical care during admission**	Diagnostics performed in the AGCH<br>Readmission to university hospital<br>Length of stay at the AGCH		R			
**Hospital readmission**	Readmission rate to the hospital or AGCH		R	R	R	R

**Health care utilization**	Home care, medical specialist care, temporary institutional care, primary care.	R		R	R	R
**Satisfaction with Care**	8 question questionnaire[53]		R	(R)		

H1= at admission, H2= at discharge, P1= one month after discharge, P3 = three months after discharge, P6 = six months after discharge. N=nurse Geriatric Community Care Hospital
P= physiotherapist D= Doctor/attending physician R= researcher/research nurse

For peer review only

For peer review only

STROBE Statement—Checklist of items that should be included in reports of ***cohort studies***

	Item No	Recommendation
**Title and abstract**	1	(*a*) Indicate the study's design with a commonly used term in the title or the abstract
		(*b*) Provide in the abstract an informative and balanced summary of what was done and what was found
**Introduction**		
Background/rationale	2	Explain the scientific background and rationale for the investigation being reported
Objectives	3	State specific objectives, including any prespecified hypotheses
**Methods**		
Study design	4	Present key elements of study design early in the paper
Setting	5	Describe the setting, locations, and relevant dates, including periods of recruitment, exposure, follow-up, and data collection
Participants	6	(*a*) Give the eligibility criteria, and the sources and methods of selection of participants. Describe methods of follow-up
		(*b*) For matched studies, give matching criteria and number of exposed and unexposed
Variables	7	Clearly define all outcomes, exposures, predictors, potential confounders, and effect modifiers. Give diagnostic criteria, if applicable
Data sources/ measurement	8*	For each variable of interest, give sources of data and details of methods of assessment (measurement). Describe comparability of assessment methods if there is more than one group
Bias	9	Describe any efforts to address potential sources of bias
Study size	10	Explain how the study size was arrived at
Quantitative variables	11	Explain how quantitative variables were handled in the analyses. If applicable, describe which groupings were chosen and why
Statistical methods	12	(*a*) Describe all statistical methods, including those used to control for confounding
		(*b*) Describe any methods used to examine subgroups and interactions
		(*c*) Explain how missing data were addressed
		(*d*) If applicable, explain how loss to follow-up was addressed
		(*e*) Describe any sensitivity analyses
**Results**		
Participants	13*	(a) Report numbers of individuals at each stage of study—eg numbers potentially eligible, examined for eligibility, confirmed eligible, included in the study, completing follow-up, and analysed
		(b) Give reasons for non-participation at each stage
		(c) Consider use of a flow diagram
Descriptive data	14*	(a) Give characteristics of study participants (eg demographic, clinical, social) and information on exposures and potential confounders
		(b) Indicate number of participants with missing data for each variable of interest
		(c) Summarise follow-up time (eg, average and total amount)
Outcome data	15*	Report numbers of outcome events or summary measures over time
Main results	16	(*a*) Give unadjusted estimates and, if applicable, confounder-adjusted estimates and their precision (eg, 95% confidence interval). Make clear which confounders were adjusted for and why they were included
		(*b*) Report category boundaries when continuous variables were categorized
		(*c*) If relevant, consider translating estimates of relative risk into absolute risk for a meaningful time period

For peer review only

For peer review only - http://bmjopen.bmj.com/site/about/guidelines.xhtml

Other analyses	17	Report other analyses done—eg analyses of subgroups and interactions, and sensitivity analyses
**Discussion**		
Key results	18	Summarise key results with reference to study objectives
Limitations	19	Discuss limitations of the study, taking into account sources of potential bias or imprecision. Discuss both direction and magnitude of any potential bias
Interpretation	20	Give a cautious overall interpretation of results considering objectives, limitations, multiplicity of analyses, results from similar studies, and other relevant evidence
Generalisability	21	Discuss the generalisability (external validity) of the study results
**Other information**		
Funding	22	Give the source of funding and the role of the funders for the present study and, if applicable, for the original study on which the present article is based

*Give information separately for exposed and unexposed groups.

Note: An Explanation and Elaboration article discusses each checklist item and gives methodological background and published examples of transparent reporting. The STROBE checklist is best used in conjunction with this article (freely available on the Web sites of PLoS Medicine at http://www.plosmedicine.org/, Annals of Internal Medicine at http://www.annals.org/, and Epidemiology at http://www.epidem.com/). Information on the STROBE Initiative is available at http://www.strobe-statement.org.

For peer review only

An acute geriatric community hospital for older adults: a study protocol for a prospective controlled observational study.

Journal:	*BMJ Open*
Manuscript ID	bmjopen-2019-033802.R1
Article Type:	Protocol
Date Submitted by the Author:	27-Jan-2020
Complete List of Authors:	Ribbink, Marthe; Amsterdam University Medical Centres, Department of Internal Medicine, Section of Geriatric Medicine<br>Macneil-Vroomen , Janet; Amsterdam University Medical Centres, Department of Internal Medicine, Section of Geriatric Medicine ; Yale School of Medicine, Department of Internal Medicine, Section of Geriatrics<br>van Seben, Rosanne; Amsterdam University Medical Centres, Department of Internal Medicine, Section of Geriatric Medicine<br>Oudejans , Irène; Amsterdam University Medical Centres, Department of Internal Medicine, Section of Geriatric Medicine<br>Buurman, Bianca; Amsterdam University Medical Centres, Department of Internal Medicine, Section of Geriatric Medicine ; Amsterdam University of Applied Sciences, ACHIEVE- Centre of Applied Research, Faculty of Health
**Primary Subject Heading**:	Geriatric medicine
Secondary Subject Heading:	Health services research, Public health
Keywords:	GERIATRIC MEDICINE, OLDER ADULTS, COMMUNITY HOSPITAL, INTERMEDIATE CARE FACILITIES, READMISSIONS, FUNCTIONAL DECLINE

SCHOLARONE™
Manuscripts

I, the Submitting Author has the right to grant and does grant on behalf of all authors of the Work (as defined in the below author licence), an exclusive licence and/or a non-exclusive licence for contributions from authors who are: i) UK Crown employees; ii) where BMJ has agreed a CC-BY licence shall apply, and/or iii) in accordance with the terms applicable for US Federal Government officers or employees acting as part of their official duties; on a worldwide, perpetual, irrevocable, royalty-free basis to BMJ Publishing Group Ltd ("BMJ") its licensees and where the relevant Journal is co-owned by BMJ to the co-owners of the Journal, to publish the Work in this journal and any other BMJ products and to exploit all rights, as set out in our licence.

The Submitting Author accepts and understands that any supply made under these terms is made by BMJ to the Submitting Author unless you are acting as an employee on behalf of your employer or a postgraduate student of an affiliated institution which is paying any applicable article publishing charge ("APC") for Open Access articles. Where the Submitting Author wishes to make the Work available on an Open Access basis (and intends to pay the relevant APC), the terms of reuse of such Open Access shall be governed by a Creative Commons licence – details of these licences and which Creative Commons licence will apply to this Work are set out in our licence referred to above.

Other than as permitted in any relevant BMJ Author's Self Archiving Policies, I confirm this Work has not been accepted for publication elsewhere, is not being considered for publication elsewhere and does not duplicate material already published. I confirm all authors consent to publication of this Work and authorise the granting of this licence.

For peer review only

An acute geriatric community hospital for older adults: a study protocol for a prospective controlled observational study.

Marthe E. Ribbink MD[1], Janet L. MacNeil Vroomen PhD[1,2], Rosanne van Seben PhD[1], Irène Oudejans MD, PhD[1], Bianca M. Buurman RN, PhD[1,3] on behalf of the AGCH-study group.

[1] Amsterdam University Medical Centre, University of Amsterdam, Department of Internal Medicine, Section of Geriatric Medicine, Amsterdam Public Health Research Institute, Meibergdreef 9, Amsterdam, The Netherlands

[2] Section of Geriatrics, Department of Internal Medicine, School of Medicine, Yale School of Medicine, The United States

[3] ACHIEVE – Centre of Applied Research, Faculty of Health, Amsterdam University of Applied Sciences, Amsterdam, The Netherlands

For peer review only

Word count, references and tables

Abstract: 268

Total word count: 4490

Number of references: 65

Number of tables/figures: 4, 2 tables and 2 figures

Appendix: STROBE checklist (at the end of the manuscript)

Email addresses authors

M.E. Ribbink, m.e.ribbink@amsterdamumc.nl

J.L MacNeil Vroomen j.l.macneil-vroomen@amsterdamumc.nl

R. van Seben rosanne.vanseben@ecorys.com

I. Oudejans i.oudejans@amc.uva.nl

B.M. Buurman b.m.vanes@amsterdamumc.nl

Corresponding author

M.E. Ribbink

Amsterdam UMC, University of Amsterdam, Department of Internal Medicine, Section of Geriatric Medicine, D3-335

PO Box 22600, 1100 DD Amsterdam, The Netherlands

For peer review only - http://bmjopen.bmj.com/site/about/guidelines.xhtml

Tel: 0031-20 5661647, Email: m.e.ribbink@amsterdamumc.nl

Keywords: geriatric medicine, older adults**,** community hospital, intermediate care facilities, readmissions, functional decline.

For peer review only

For peer review only - http://bmjopen.bmj.com/site/about/guidelines.xhtml

Abstract

Introduction: Hospital admission is associated with unwanted outcomes like readmission, institutionalization, functional decline, and mortality in older adults with multiple chronic conditions. Providing acute care in the community and integrating effective components of care models might lead to a reduction of negative outcomes. Recently, the first geriatrician-led care Acute Geriatric Community Hospital (AGCH) was introduced in the Netherlands. Care at the AGCH is focused on: treatment of acute disease, comprehensive geriatric assessment, setting patient-led goals, early rehabilitation and stream-lined transitions of care.

Methods and analysis: This prospective cohort study will investigate the effectiveness of care delivery at the AGCH on patient outcomes, by comparing AGCH patients to two historic cohorts of hospitalized patients. Propensity score matching will correct for potential population differences. The primary outcome is the three-month unplanned readmission rate. Secondary outcomes include: functional decline, institutionalization, healthcare utilization, occurrence of delirium or a fall, health-related quality of life, mortality and patient satisfaction. Measurements will be conducted at admission, discharge and one, three and six months after discharge. Furthermore, an economic evaluation and qualitative process evaluation to assess facilitators and barriers for implementation is planned.

Ethics and dissemination: The study will be conducted according to the Declaration of Helsinki. The Medical Ethics Research Committee (METC) confirmed that the Medical Research Involving Human Subjects Act did not apply to this research project and official approval was not required. The findings of this study will be disseminated through academic and public lectures, scientific conferences and in peer-reviewed journals. Furthermore, the findings of this study will aid in the implementation and financing of this concept (inter)nationally.

Trial Registration Number NL7896; pre-results

For peer review only - http://bmjopen.bmj.com/site/about/guidelines.xhtml

Strengths and limitations of this study:

- This study will be the first to evaluate an acute geriatric community hospital in the Netherlands on both patient reported and economic outcomes.
- Patients, informal caregivers and professionals were involved in the design, and implementation of the Acute Geriatric Community Hospital.
- A process evaluation is planned to describe the experience of various stakeholders with this new concept and reveal barriers and facilitators to its' implementation.
- A limitation of this study is the use of two historic cohorts as control population, as this may result in baseline differences between the control and intervention population

For peer review only

For peer review only - http://bmjopen.bmj.com/site/about/guidelines.xhtml

Introduction

Background

Throughout the western world, there is an increase of older adults requiring acute care. Inpatient services are mostly consumed by those over the age of 65.[1,2] The Netherlands, like many other countries, recently (2015) implemented stay-at-home policies leading to an increase of frail older persons living longer in the community.[3] These reforms juxtaposed with an increased aging population, contribute to increased acute care utilization.[4] There has been a 19% increase in emergency department visits by Dutch older adults based on data from 2015 versus 2017.[5,6]

Many older adults come to the hospital with complex and atypical health problems.[5,7] When older persons are subsequently hospitalized, health outcomes are known to be poor,[8] particularly in patients with geriatric syndromes such as cognitive impairment or mobility impairment.[9,10] For example, previous research showed that 30% of older persons gained new disabilities and 20% were readmitted within 30 days postdischarge.[11,12] Hospitalization itself may contribute to these poor outcomes as hospitalized older adults often have reduced mobility while bedbound for approximately 20 hours daily.[13,14] Low physical activity, in combination with poor nourishment and increased caloric demand due to acute illness can lead to loss of muscle mass and may contribute to the development of new disabilities, particularly in frail patients.[15,16] Together with noise in a hospital environment and different personnel rotating through patient rooms, this contributes to sensory overstimulation and sleep deprivation, which may lead to confusion and the occurrence of delirium.[17,18,19] Not only is the patient affected during hospitalization, informal caregivers also find hospital admissions stressful.[20] Furthermore, previous research shows that lack of discharge planning in the hospital can result in the care needs of patients being unmet.[21] Hospital care as usual compared to discharge planning and follow-up showed an increase in early readmissions.[22] Readmissions can further effect patients' recovery and increase healthcare costs.[23]

For peer review only - http://bmjopen.bmj.com/site/about/guidelines.xhtml

The complex medical needs of older persons, combined with their more dependent social situation requires care delivery that offers guidance and support of realistic health and life goals.[24] Perhaps a 'gap' exists between what care can be provided in an acute care hospital, that is secondary care, with a focus on medical treatment and diagnostics, versus what can be provided in the community, that is primary care focused on rehabilitation, nursing care and wellbeing.

Several alternative strategies to hospital admission and (nurse-led) intermediate care have been developed in the past as a substitute to conventional hospitalization.[25] Examples include (nurse-led) intermediate care and subacute geriatric care units, low-tech but with geriatric expertise.[26,27] In general, these types of care have comparable outcomes to hospital care as usual. Moreover, nurse-led care in the US, observation units and hospital at home care all showed a cost reduction compared to care as usual.[25,26] Until recently, the Netherlands however, had limited alternatives to hospitalization for older persons who required acute care. Therefore, our research group sought to create an acute care alternative and opened the Acute Geriatric Community Care Hospital (AGCH) in July 2018, partnering with an academic hospital (Amsterdam UMC, location AMC), an insurance company (Zilveren Kruis) and a home care and nursing home agency (Cordaan). This acute geriatric care unit, which is based within an intermediate care facility, provides an alternative to conventional hospitalization and delivers acute care closer to home.

The AGCH delivers acute care that is focused on early mobilization and rehabilitation. Older persons with common medical problems (such as urinary tract infections, pneumonia or heart failure) and geriatric syndromes requiring hospital admission can be admitted to the AGCH. The AGCH provides a form of *intermediate* care between primary and secondary care. In the Netherlands, primary care includes general practice, community nursing and (temporary) admission to nursing home. Secondary care includes medical specialist care and hospital admission. Care is supervised by a geriatrician and provided by nurses trained in geriatric care who have experience as either a hospital or community nurse. The single rooms are designed to accommodate respite for the informal

For peer review only - http://bmjopen.bmj.com/site/about/guidelines.xhtml

caregivers. This concept of care is new to the Netherlands, to our knowledge there is only one example in Europe to which it compares: a "subacute care unit" in intermediate care, which has been implemented in Spain.[27]

Our hypothesis is that with the provision of integrated medical and nursing care close to home, the AGCH is better suited to the needs of older adults with multiple chronic conditions and will lead to better patient health outcomes and reduced post-acute care costs. Therefore, this study is designed to compare care provided for older patients in the AGCH versus care provided in a hospital setting. Specifically we aim to:

- Evaluate the 90-day readmission rate of patients acutely admitted to AGCH compared to a traditional hospital (usual care). Secondary outcomes include: functional decline, institutionalization, healthcare utilization, the occurrence of geriatric syndromes such as delirium, health-related quality of life, mortality, and patient satisfaction;
- Assess the cost-effectiveness of the AGCH versus usual care by performing an economic evaluation from a health care provider and societal perspective;
- Conduct a process evaluation using interviews with key stakeholders to identify facilitators and barriers to the implementation of the AGCH.

Methods

Setting

The Acute Geriatric Community Hospital opened in July 2018. It serves both the south-eastern part of Amsterdam and its surrounding areas (an area with approximately 147 500 inhabitants).[28] The AGCH is a 23 -bed facility within an skilled nursing facility. The hospital has 24-hour geriatric and nursing assistance, physiotherapy and routine laboratory testing during the workweek and simple x-ray available once a week. The population that is eligible for admission to the AGCH includes patients with 1) acute medical problems requiring hospitalization (e.g. pneumonia, exacerbation of heart

For peer review only - http://bmjopen.bmj.com/site/about/guidelines.xhtml

failure or an urinary tract infection) 2) geriatric conditions (e.g. delirium, cognitive impairment, falls, functional impairment), who are 3) hemodynamically stable and 4) not in need of complex diagnostic testing. In general, patients will not be admitted if they: 1) require care that can only be provided at an intensive care unit 2) require surgery 3) require urgent treatments or diagnostic tests that can only be provided in-hospital (e.g. endoscopy, interventional radiology) 4) do not need hospital care, but require transfer to a skilled nursing facility and 5) live in another region of the Netherlands. Patients are directly admitted to the AGCH from the emergency department (ED) of the Amsterdam UMC- location Academic Medical Centre (AMC) in Amsterdam which is a 1000-bed academic hospital with approximately 30,000 ED visits yearly. After the on-call geriatrician has assessed that patient is eligible for AGCH admission and the patient or representative has agreed to admission, the patient is transferred to the AGCH by ambulance. Since October 2019 patients can also be transferred from EDs of other hospitals in Amsterdam. In the future we plan to admit patients from home or a General Practice office. Patients are admitted between 8.00 am and 11.00 pm, 7 days a week. At admission, a Comprehensive Geriatric Assessment (CGA) is conducted.[29] The CGA gives an overview of all medical, functional, psychological and social problems that are discussed during multidisciplinary team meetings and are used to formulate a care plan for each patient. For an overview of the admission process, admission criteria and components of this intervention see figure 1.

Study design

This study is a prospective, observational, cohort study with two historical control groups to evaluate the clinical and economic effects of the AGCH. The STROBE statement was used in preparing the study protocol.[30] (Appendix 1) Participants will be compared to hospital controls. The participants are recruited into the study and are assessed at admission, discharge, one month, three and six months after discharge. The recruitment phase of this study started in February 2019. We plan to recruit for 18 to 24 months. The first three months of data collection will consist of a piloting phase to assess the feasibility of data-collection and follow-up. In addition, a qualitative process evaluation

For peer review only - http://bmjopen.bmj.com/site/about/guidelines.xhtml

on facilitators and barriers to the implementation of the AGCH and patient experience will be conducted.

Participants

Patients admitted to the AGCH are eligible for inclusion to the study. However, patients are excluded from the study if: 1) the attending physician judges that the patient is too ill to participate e.g. is terminally ill 2) the patient or legal representative does not consent to participate. 3) the patient or legal representative does not speak or understand Dutch or English. In the case of cognitively impaired or delirious patients, patients can only be included if a legal representative consents to participation and acts as healthcare-proxy. Cognitive functioning is assessed by the attending physician and confirmed by the researcher by conducting a Mini Mental State Exam (MMSE). A MMSE score of 15 or less Indicates severe cognitive impairment, in which the approval of a legal representative will be sought.[31]

Historical control groups

Two completed studies conducted by our research group were selected as historical control groups. These control groups were selected based on characteristics of the participants -primary admission diagnosis, department, area of residence- and the availability and reproductively of the data. The first control group from the Transitional Care Bridge Study consists of 674 patients that were recruited between September 2010 and March 2014 originating from the greater Amsterdam area in the Netherlands.[32] Participants were patients of 65 years and older admitted for at least 48 hours to an internal medicine ward. Proxy consent was provided for participants suffering from severe cognitive impairment (Mini Mental State Exam[31] (MMSE) ≤15). They participated in a negative randomized controlled trial that assessed the effectiveness of a nurse-led transitional care program in preventing functional decline.[32] The second control group (Hospital-ADL study[10]) consists of 401

For peer review only - http://bmjopen.bmj.com/site/about/guidelines.xhtml

patients that were recruited between October 2015 and June 2017, also originating from the greater Amsterdam area. These participants were enrolled in a prospective cohort studying the trajectory of functional decline in older hospitalized adults. Participants were aged 70 years and older, hospitalized for at least 48 hours. Patients suffering from severe cognitive impairment (MMSE ≤15) and delirium were excluded from participation. For the detailed methodology and inclusion criteria of the two control cohorts we refer to the study protocols and papers of these studies.[10,32-34]

Patient and public involvement

Older persons living in Amsterdam were involved in the design of the AGCH concept. There was no patient involvement in the design of this study.

Outcomes

The primary outcome measure is the 3-month unplanned readmission rate to the AGCH or hospital. Secondary outcomes measured at 1,3 and 6 months will include:

1) ADL-functioning as defined by the KATZ-ADL scale.[35]

2) Healthcare utilization, including institutionalization in a long-term care facility.

3) Occurrence of delirium and/or falls.

4) Health-related quality of life (HRQoL).[36]

5) All-cause mortality.

6) Satisfaction of patients and primary care givers with the care provided.

Data collection

Eligible patients and/or legal representatives will be contacted and informed about the study procedures after which written informed consent is obtained. Inclusion and interviewing of patients is conducted by an onsite researcher. Routine data on functioning and risk assessments are collected

For peer review only - http://bmjopen.bmj.com/site/about/guidelines.xhtml

by a trained registered nurse and physiotherapist as part of the CGA for each patient.[37] Table 1 gives an overview of measurement of primary and secondary outcomes over time. These measurements were chosen based on the assessments and data collected from the two historic control groups. Measurements during admission are at H1 which is within 48 hours after admission and H2 which is within 48 hours before discharge. Follow-up is completed by telephone at 1,3 and 6 months after discharge (P1, P3 and P6).

Data collection includes:

1. Medical and demographical data

Sociodemographic data. These will include age, gender, highest level of education, ethnicity, marital status and living arrangement.

Time spent at the ED, admission diagnosis, date and time of admission.

Chronic conditions. The number and severity of chronic conditions will be assessed using the Charlson Comorbidity Index.[38] This index is commonly used to indicate the risk of mortality; each condition is scored 1, 2, 3 or 6 points, with a higher total number of points indicating a greater risk at death.

Polypharmacy. Polypharmacy will be assessed by counting the number of individual drugs that are chronically prescribed to a participant, in which a number of 5 or more drugs is considered polypharmacy.

Mortality. This will be assessed during follow-up, where possible by reviewing patients electronic files, otherwise from general practice registries.

2. Cognitive functioning.

Cognitive impairment. This will be is assessed by reviewing the score of the Mini Mental State Exam (MMSE) that is performed within 48 hours of admission. The MMSE includes 23 items (total score 0-

For peer review only

30) that screen for cognitive impairment. A score of 23 or less is defined as possible cognitive impairment.[31] When a patient is delirious upon inclusion the MMSE is not conducted.

Delirium. The Confusion Assessment Method (CAM), the short 4 item version, is used to assess the presence and duration of delirium.[39] The CAM is widely used by physician and nurse practitioners to diagnose delirium (sensitivity of 53-90% and specificity of 84-100%).[40] The CAM is filled out within 24 hours of admission. Moreover, the risk on developing delirium is assessed using the Dutch VMS criteria for risk on delirium.[41] Nurse practitioners will score the CAM daily from day one till day three of admission, if there are signs of possible delirium at day 3, these measurements are continued until the symptoms are resolved. In addition, during the first three days of admission the Delirium Observation Screening Scale (DOSS) is scored during each nursing shift and is continued when there is a clinical suspicion of delirium.[42]

3. Psychosocial functioning and quality of life

Apathy. We use three items of the *Geriatric Depression Scale* (GDS-15) to assess apathy (sensitivity of 69% and specificity of 85 %). These items include the following questions: 1) 'Do you prefer to stay at home, rather than going out and doing new things' 2) 'Have you dropped many of your activities and interests?' And 3) 'Do you feel full of energy'. A score of >2 points is classified as 'apathy present'. [43]

Social network and informal care. Participants are asked if they receive informal care, how many hours a week, what type of care (housekeeping and/or personal care) and from which persons (partners, children, other family members or neighbours/volunteers).

Health-Related Quality of Life. This will be measured by determining Health-Related Quality of Life using the EuroQoL-5D (EQ-5D). The EQ-5D is a broadly used and validated instrument for measuring generic health-related quality of life.[36]

For peer review only - http://bmjopen.bmj.com/site/about/guidelines.xhtml

4. Physical functioning

Risk of functional decline. Patients are assessed for risk of functional decline using the Identification of Seniors at Risk- Hospitalized patients (ISAR-HP), scores of two and up indicate at an increased risk for functional decline.[44]

Functioning level. The 15-item modified KATZ score is used to measure Activities of Daily Living (ADL)-functioning. This included assessment of performance in basic ADL- KATZ-6), as in instrumental ADL (KATZ-9).[45,46] We measure KATZ-score both currently (at admission), as two weeks before admission, reflecting pre-morbid level of functioning. (I)ADL-functional is also included in follow-up measurements.

(Im)mobility. We will assess mobility by reviewing three questions that are in the admission assessment regarding: 1) use of a walking aid (from KATZ-15), and from the CGA: 2) being able to walk outside of the house for five minutes (two weeks before and currently) and 3) performing, and frequency of, physical activity.[47]

Handgrip strength. Physiotherapists measure muscle weakness in all admitted patients using maximum handgrip strength (JAMAR). [48]

Gait speed. Gait speed is measured as part of the Short Physical Performance Battery (SPBB) that is part of the physiotherapist' admission assessment.[49]

Falls. Fall history is assessed by asking the number of falls in the past six months[41] During the discharge assessment the occurrence of falls in the AGCH and the consequences of falls (indication for prolonged stay, diagnostics or injury) are recorded.

Fear of falling. We will use a Numeric Rating Scale (NRS, score 0-10) to assess fear of falling, 0 indicates no fear of falling, and 10 the greatest fear of falling possible.[34]

For peer review only - http://bmjopen.bmj.com/site/about/guidelines.xhtml

Pain. Widely used in clinical practice the standard for pain assessment is the Numeric Rating Scale, ranging from 0 to 10, in which a score of 0 represents no pain and 10 represents the worst possible pain.[50]

Fatigue. A Numeric Rating Scale from 0-10 is used, 0 indicating no fatigue and 10 indicating the greatest fatigue ever felt by the participant.[51]

Sleep. Participants are asked if they have had difficulties with sleeping in the past month and whether participants have used sleep medication.

Nutrition. We will use the Short Nutritional Assessment Questionnaire (SNAQ) for identifying malnourished patients. The SNAQ consists of three questions on weight loss, appetite and drink/tube nutrition, resulting in a score ranging from 0 to 5. 0 and 1 are defined as 'no malnutrition' 2 as 'moderate malnutrition' and 3 or more as 'severe malnutrition'.[52]

5. Healthcare utilization and satisfaction with care

Medical care during admission and process of discharge. The following are collected from patient electronic health records: the diagnostics performed in the AGCH, revisits to the hospital, admission to the hospital, length of stay of the AGCH, discharge destination and time needed to send medical handovers to the general practitioner.

Hospital readmission. This outcome will be assessed during follow-up. Follow-up will consist of three telephone interviews at 1, 3 and 6 months after discharge. Readmission will be both assessed by interview as by checking care data from an aggregated database of expense claims of various healthcare insurers. Data that will be collected are: number of readmissions, total days of readmission, reasons for readmission and whether the readmission was planned or unplanned.

Emergency department (ED) visits. ED visits will be assessed during follow-up and checked in insurance data. We will record the number of separate ED visits.

For peer review only

For peer review only - http://bmjopen.bmj.com/site/about/guidelines.xhtml

Outpatient hospital visits. We will ask patients if there have been any outpatient visits in the past month(s), and if so how many.

Consultations by general practitioner. We will ask patients if there have been any consultations by the general practitioner (both during the day as during out-of-office hours) visits in the past month(s), and if so how many.

Consultations by physiotherapist or dietician. We will ask patients if there have been any consultations by a physiotherapist of dietician in the past month(s), and if so how many.

Home care. This includes questions on frequency of home care, including housekeeping, personal care and nursing care. We will also include days of day care and hours of informal care provided by family members or friends.

Temporary admission to a nursing home. Days of (temporary) admission to a skilled nursing facility or rehabilitation facility.

Permanent Institutionalization. This concerns long-term admission to a skilled nursing facility and date of admission to this facility.

Patient satisfaction with care. Patients or informal caregivers are asked to fill out an 8-question questionnaire regarding their satisfaction with the care that they received. Questions are answered on a 5- level Likert scale.[53]

Sample Size calculation

In the Hospital-ADL study 34 % of participants experienced a readmission at 90 days.[34] Assuming that 26% of patients admitted to the AGCH will experience a 90 day readmission, data from 515 patients of AGCH will yield 80% power to detect an absolute difference of 8% in readmission rate (which is a 25% reduction in relative risk) using a two-sided test with an alpha of 0.05.[54] As we expect 10% loss to follow-up, we will aim to include a total of 567 (= 515*1.10) patients from the AGCH.

For peer review only - http://bmjopen.bmj.com/site/about/guidelines.xhtml

Planned statistical analysis

The complete participant flow diagram will show a summary of admissions and study recruitment at the AGCH and will provide study discontinuation rates at 1, 3 and 6 months follow-up.[30] We will describe demographic, clinical and prognostic characteristics of the study participants at baseline. The number of participants with missing data will be collected and described alongside our variables to check for the pattern of missingness. Inversely-weighted propensity scores will be used to control for any imbalances between the treatment groups.[55] Propensity scores will be calculated using generalized booted methods. Balance and overlap of propensity scores distribution will be assessed. Propensity score weights for the estimation of the average treatment effect will be created using all covariates where groups differed on baseline or that were associated with 90-day readmission rate. As this is a repeated measures design, we will assume equal weighting for all measurements.[56]

All hypotheses will be tested using two-tailed- significance level of 0.05. All secondary outcomes will be adjusted for multiple testing using a Hochberg method.[57,58] Descriptive analyses will be performed to examine participant's characteristics. Differences in changes over time in outcomes will be compared between groups using multilevel models. All models will include a main effect of treatment group, a linear term for time and an interaction between time and treatment group. Models will be checked with residual and appropriate goodness-of-fit statistics.

Economic evaluation

A healthcare and societal perspective is planned for this economic evaluation. The evaluation from the healthcare perspective will only include direct medical costs accrued in the six months after the admission to the AGCH. Direct medical cost will only include costs that are funded through the Dutch healthcare system. The evaluation from a societal perspective will include an estimation of the cost of informal care. Costs will be based on the reference prices found in the Dutch Manual for Costing studies and will be set for final year of data collection (2020 or 2021). According to this guideline

For peer review only - http://bmjopen.bmj.com/site/about/guidelines.xhtml

costs will be discounted at 4% and quality adjusted life years (QALYs) will be discounted at 1,5 %.[59] Propensity scores will also be used in the economic evaluation. Missing data will be imputed using multiple imputation chained equations if necessary, for cost and effect data. We plan to use generalized linear regression models with a gamma distribution and an identity link to account for the right skew of cost data. A generalized linear regression model will be used to estimate the incremental effect in QALYs. adjusted for baseline utility estimates with a Gaussian distribution and identify link.[60] Incremental cost-effectiveness ratios will be calculated using the pooled cost and effect estimates. Bootstrapped cost-effect pairs will be plotted on a cost-effectiveness plane and used to estimate cost-effectiveness acceptability curves.[61]

Process evaluation and patient experience

We plan to use a qualitative study design to describe barriers and facilitators to implementation of the AGCH- concept and describe experiences of patients and healthcare professionals with the AGCH. We will conduct semi-structured interviews with various stakeholders, such as geriatricians, nurses, physiotherapists and hospital administrators. These interviews will concern the implementation of the AGCH concept. In addition, semi-structured interviews with patients and informal caregivers will be conducted in order to describe the patient experience and satisfaction with this new form of care. A representative sample of patients and/or caregivers who participate in the prospective cohort study will approached and invited to be interviewed shortly after the discharge from the AGCH. Stakeholders and healthcare professionals will be selected by a researcher and will be invited for an interview to discuss their experience and opinion on the AGCH. Interviews will be typed verbatim and analysed by two researchers independently, using thematic analysis.[62] In our analysis of barriers and facilitators to implementation, we will describe these factors at different levels: micro (healthcare professionals), meso (care organizations) and macro level (legal and financial framework).[63] The findings will be summarized in matrices with facilitators and barriers at different

For peer review only

For peer review only - http://bmjopen.bmj.com/site/about/guidelines.xhtml

levels (micro, meso, macro) and can be used to develop a guideline for implementation of the AGCH elsewhere.[64]

Preliminary results

Between February 1st and December 20th 2019 there were 362 consecutive admissions to the AGCH. Of these admissions 26 were readmissions of patients who were already study participants. Of the remaining 336 admissions 90 were by patients who did not meet the inclusion criteria. Of the remaining patients 246 patients or legal representatives and healthcare-proxy were approached for participation; 212 consented to participation. (figure 2) The healthcare –proxy provided informed consent in 62 (29.2 %) of cases. 16 patients did not consent to follow-up by telephone but did consent to medical record review. The total study sample as of December 20th 2019 consisted of 212 participants at baseline. Table 2 displays the baseline characteristics of this group. Participants had a mean age (standard deviation) of 81.8 (8.4) years, 47.6 % were male. Most participants were living independently before admission (81.1%). Most frequent admission diagnosis were infectious diseases (28.3%, mostly urinary tract infections), respiratory-related (25.5%, including pneumonia which was over half respiratory-related and exacerbations of COPD), and other (geriatric) diagnoses such as falls, delirium or sudden unexplained functional decline (30.2%). Cardiac (9.4%) admission diagnosis concerned mostly exacerbations of heart failure. Median length of stay was (interquartile range) 8.0 days (5.0-12.0) and 83.7 % were discharged to their original living situation.

Discussion

The complex acute medical needs of older patients require the delivery of specialized geriatric care. The traditional hospital environment may however not support recovery and maintaining

For peer review only - http://bmjopen.bmj.com/site/about/guidelines.xhtml

independence. The AGCH aims to deliver care that focusses both on medical treatment, early rehabilitation and proper transitions of care for older adults with multiple chronic conditions.[29,65] The AGCH is unique in the Netherlands in its aim to combine multiple evidenced-based components of care for frail older persons at an alternative location for hospital care. The proposed research will provide insight into the clinical and economic effectiveness of care delivered at the AGCH, compared to hospital care.

Our preliminary results show that data collection at the AGCH is feasible and we expect to recruit enough patients to evaluate the primary outcome. There are also limitations to the design of this study. It is a non-randomized study and that historic cohorts are used as control groups. Therefore baseline differences between intervention and control groups may hamper the matching between the groups. Also, as the data from the cohorts were not collected in the same time period as the AGCH cohort there may be external non-observed differences in the Dutch healthcare system and work processes in hospitals may have changed over the years. However, the two control populations were not self-selected and do represent a geriatric population suffering from common exacerbations of chronic conditions and acute illness that occur in older persons. Strengths are that patients and informal caregivers were involved in the design of the concept of the AGCH. Moreover, a process evaluation will address the barriers and facilitators to implementation of a community hospital such as the AGCH in the existing health care system of the Netherlands. This research will provide valuable insights into the implementation of this concept of care in other regions of the Netherlands and abroad.

Ethics and Dissemination

This study will be carried out in accordance with the declaration of Helsinki and current ethical requirements. The outcomes of this study will be reported according to STROBE guidelines for cohort

For peer review only

studies.[30] This study will evaluate both the effectiveness of this type of care delivery as of the costs that are involved, allowing for implementation elsewhere. The findings of this study will be published in peer-reviewed journals.

Acknowledgements

The authors thank all (care) professionals from the Amsterdam University Medical Centres, Cordaan and Zilveren Kruis who have worked on the development of the AGCH. Thank you for your time, advise and cooperation. Also, we would like to thank the members of the AGCH-study group, these are the clinicians who work at the Geriatrics Department of the Amsterdam University Medical Centres and who support the data-collection at the AGCH.

Author contributions

MER, JMV and RS contributed to the design of the protocol and drafting of the manuscript. IO and BMB made substantial contributions to the design and clinical aspects of the of the protocol. BMB conceived the study and wrote funding applications. All authors critically revised the manuscript and approved the final version of this manuscript.

Funding statement

This research received funding though ZonMw, the Netherlands Organization for Health Research and Development, project number 808393598041. The care provided at the AGCH (WijkKliniek) is provided in a partnership between Cordaan, a community and home-care organization and the Amsterdam University Medical Centres, location Academic Medical Centre. The AGCH (WijkKliniek) is financially supported by Zilveren Kruis, a health insurance company.

Patient consent

Not required.

Competing interests statement- None Declared

Ethics approval

Based on the study protocol, the Ethics Committee (METC) of the Amsterdam University Medical Center waived the obligation for the study to undergo formal ethical approval as is described under Dutch law in the Medical Research in Humans Act, January 2019. (ref W17_474 # 19.001) As this is a prospective study and pseudonymized data is used written informed consent was obtained from the participants prior participation. This is in line with current European legislation under the General Data Protection Regulation (GDPR).

References

1. McCabe JJ, Kennelly SP. Acute care of older patients in the emergency department: strategies to improve patient outcomes. *Open Access Emerg Med.* 2015;7:45-54.
2. Centraal Bureau voor de Statistiek. Ziekenhuisopnamen; kerncijfers; geslacht, leeftijd en regio. http://statline.cbs.nl/StatWeb/publication/?DM=SLNL&PA=71857ned. Published 2014. Accessed.

For peer review only - http://bmjopen.bmj.com/site/about/guidelines.xhtml

3. Government of the Netherlands Ministry of Health Welfare and Sport- Living Independently for Longer https://www.government.nl/topics/care-and-support-at-home/living-independently-for-longer. Published 2019. Accessed.

4. Jayaprakash N, O'Sullivan R, Bey T, Ahmed SS, Lotfipour S. Crowding and delivery of healthcare in emergency departments: the European perspective. *West J Emerg Med.* 2009;10(4):233-239.

5. de Kousemaeker G, Vandermeulen L. *Ontwikkelingen Langdurige Zorg* Fluent Healthcare Amsterdam 2017.

6. DeFrances CJ, Lucas CA, Buie VC, Golosinskiy A. 2006 National Hospital Discharge Survey. *National health statistics reports.* 2008(5):1-20.

7. Boyd CM, Xue QL, Guralnik JM, Fried LP. Hospitalization and development of dependence in activities of daily living in a cohort of disabled older women: the Women's Health and Aging Study I. *The journals of gerontology Series A, Biological sciences and medical sciences.* 2005;60(7):888-893.

8. Buurman BM, Han L, Murphy TE, et al. Trajectories of Disability Among Older Persons Before and After a Hospitalization Leading to a Skilled Nursing Facility Admission. *Journal of the American Medical Directors Association.* 2016;17(3):225-231.

9. Fogg C, Griffiths P, Meredith P, Bridges J. Hospital outcomes of older people with cognitive impairment: An integrative review. *International journal of geriatric psychiatry.* 2018.

10. van Seben R, Reichardt LA, Aarden J, et al. Geriatric syndromes from admission to 3 months post-discharge and their association with recovery. *Innovation in Aging.* 2017;1(suppl_1):902-903.

11. Boyd CM, Landefeld CS, Counsell SR, et al. Recovery of activities of daily living in older adults after hospitalization for acute medical illness. *Journal of the American Geriatrics Society.* 2008;56(12):2171-2179.

12. Kansagara D, Englander H, Salanitro A, et al. Risk prediction models for hospital readmission: a systematic review. *Jama.* 2011;306(15):1688-1698.

13. Brown CJ, Redden DT, Flood KL, Allman RM. The underrecognized epidemic of low mobility during hospitalization of older adults. *Journal of the American Geriatrics Society.* 2009;57(9):1660-1665.

14. Agmon M, Zisberg A, Gil E, Rand D, Gur-Yaish N, Azriel M. Association Between 900 Steps a Day and Functional Decline in Older Hospitalized Patients. *JAMA internal medicine.* 2017;177(2):272-274.

15. Kortebein P, Symons TB, Ferrando A, et al. Functional impact of 10 days of bed rest in healthy older adults. *The journals of gerontology Series A, Biological sciences and medical sciences.* 2008;63(10):1076-1081.

16. Zisberg A, Shadmi E, Gur-Yaish N, Tonkikh O, Sinoff G. Hospital-associated functional decline: the role of hospitalization processes beyond individual risk factors. *Journal of the American Geriatrics Society.* 2015;63(1):55-62.

17. Missildine K, Bergstrom N, Meininger J, Richards K, Foreman MD. Sleep in hospitalized elders: a pilot study. *Geriatric nursing (New York, NY).* 2010;31(4):263-271.

18. Missildine K, Bergstrom N, Meininger J, Richards K, Foreman MD. Case studies: is the sleep of hospitalized elders related to delirium? *Medsurg Nurs.* 2010;19(1):39-46.

19. Dasgupta M, Brymer C. Poor functional recovery after delirium is associated with other geriatric syndromes and additional illnesses. *International psychogeriatrics.* 2015;27(5):793-802.

20. Rodakowski J, Rocco PB, Ortiz M, et al. Caregiver Integration During Discharge Planning for Older Adults to Reduce Resource Use: A Meta-analysis. *Journal of the American Geriatrics Society.* 2017.

21. Holland DE, Mistiaen P, Bowles KH. Problems and unmet needs of patients discharged "home to self-care". *Professional case management.* 2011;16(5):240-250; quiz 251-242.

For peer review only - http://bmjopen.bmj.com/site/about/guidelines.xhtml

22. Naylor MD, Brooten D, Campbell R, et al. Comprehensive discharge planning and home follow-up of hospitalized elders: a randomized clinical trial. *Jama.* 1999;281(7):613-620.
23. Mechanic R. Post-acute care--the next frontier for controlling Medicare spending. *The New England journal of medicine.* 2014;370(8):692-694.
24. Westphal EC, Alkema G, Seidel R, Chernof B. How to Get Better Care with Lower Costs? See the Person, Not the Patient. *Journal of the American Geriatrics Society.* 2016;64(1):19-21.
25. Conley J, O'Brien CW, Leff BA, Bolen S, Zulman D. Alternative Strategies to Inpatient Hospitalization for Acute Medical Conditions: A Systematic Review. *JAMA internal medicine.* 2016;176(11):1693-1702.
26. Griffiths PD, Edwards MH, Forbes A, Harris RL, Ritchie G. Effectiveness of intermediate care in nursing-led in-patient units. *The Cochrane database of systematic reviews.* 2007(2):CD002214.
27. Inzitari M, Gual N, Roig T, et al. Geriatric Screening Tools to Select Older Adults Susceptible for Direct Transfer From the Emergency Department to Subacute Intermediate-Care Hospitalization. *Journal of the American Medical Directors Association.* 2015;16(10):837-841.
28. Stadindex. Stadindex- Noord- Holland https://www.stadindex.nl/noord-holland. Published 2019. Accessed 11th of June 2019
29. Stuck AE. [Multidimensional geriatric assessment in the acute hospital and ambulatory practice]. *Schweizerische medizinische Wochenschrift.* 1997;127(43):1781-1788.
30. von Elm E, Altman DG, Egger M, et al. The Strengthening the Reporting of Observational Studies in Epidemiology (STROBE) statement: guidelines for reporting observational studies. *Journal of clinical epidemiology.* 2008;61(4):344-349.
31. Folstein MF, Folstein SE, McHugh PR. "Mini-mental state". A practical method for grading the cognitive state of patients for the clinician. *Journal of psychiatric research.* 1975;12(3):189-198.
32. Buurman BM, Parlevliet JL, Allore HG, et al. Comprehensive Geriatric Assessment and Transitional Care in Acutely Hospitalized Patients: The Transitional Care Bridge Randomized Clinical Trial. *JAMA internal medicine.* 2016;176(3):302-309.
33. Buurman BM, Parlevliet JL, van Deelen BA, de Haan RJ, de Rooij SE. A randomised clinical trial on a comprehensive geriatric assessment and intensive home follow-up after hospital discharge: the Transitional Care Bridge. *BMC health services research.* 2010;10:296.
34. Reichardt LA, Aarden JJ, van Seben R, et al. Unravelling the potential mechanisms behind hospitalization-associated disability in older patients; the Hospital-Associated Disability and impact on daily Life (Hospital-ADL) cohort study protocol. *BMC geriatrics.* 2016;16(1):59.
35. Katz S. Assessing self-maintenance: activities of daily living, mobility, and instrumental activities of daily living. *Journal of the American Geriatrics Society.* 1983;31(12):721-727.
36. EuroQol--a new facility for the measurement of health-related quality of life. *Health policy (Amsterdam, Netherlands).* 1990;16(3):199-208.
37. Ellis G, Whitehead MA, Robinson D, O'Neill D, Langhorne P. Comprehensive geriatric assessment for older adults admitted to hospital: meta-analysis of randomised controlled trials. *BMJ (Clinical research ed).* 2011;343:d6553.
38. Charlson ME, Pompei P, Ales KL, MacKenzie CR. A new method of classifying prognostic comorbidity in longitudinal studies: development and validation. *Journal of chronic diseases.* 1987;40(5):373-383.
39. Inouye SK, van Dyck CH, Alessi CA, Balkin S, Siegal AP, Horwitz RI. Clarifying confusion: the confusion assessment method. A new method for detection of delirium. *Annals of internal medicine.* 1990;113(12):941-948.
40. NVKG-Dutch-Society-for-Clinical-Geriatrics. Meetinstrumenten voor diagnose delier. https://richtlijnendatabase.nl/richtlijn/delier_bij_volwassenen/meetinstrumenten_voor_diagnose_delier.html#onderbouwing. Published 2013. Accessed 8th of January 2019.
41. Heim N, van Fenema EM, Weverling-Rijnsburger AW, et al. Optimal screening for increased risk for adverse outcomes in hospitalised older adults. *Age and ageing.* 2015;44(2):239-244.

For peer review only

For peer review only - http://bmjopen.bmj.com/site/about/guidelines.xhtml

42. Schuurmans MJ, Shortridge-Baggett LM, Duursma SA. The Delirium Observation Screening Scale: a screening instrument for delirium. *Res Theory Nurs Pract.* 2003;17(1):31-50.

43. van der Mast RC, Vinkers DJ, Stek ML, et al. Vascular disease and apathy in old age. The Leiden 85-Plus Study. *International journal of geriatric psychiatry.* 2008;23(3):266-271.

44. Hoogerduijn JG, Schuurmans MJ, Duijnstee MS, de Rooij SE, Grypdonck MF. A systematic review of predictors and screening instruments to identify older hospitalized patients at risk for functional decline. *Journal of clinical nursing.* 2007;16(1):46-57.

45. Katz S, Downs TD, Cash HR, Grotz RC. Progress in development of the index of ADL. *The Gerontologist.* 1970;10(1):20-30.

46. Laan W, Zuithoff NP, Drubbel I, et al. Validity and reliability of the Katz-15 scale to measure unfavorable health outcomes in community-dwelling older people. *The journal of nutrition, health & aging.* 2014;18(9):848-854.

47. Stichting-effectieve-ouderenzorg. Comprehensive Geriatric Assesment TZB https://effectieveouderenzorg.nl/zorgmodellen/comprehensive-geriatric-assessment/. Published 2013. Accessed 8th of January 2019.

48. Roberts HC, Denison HJ, Martin HJ, et al. A review of the measurement of grip strength in clinical and epidemiological studies: towards a standardised approach. *Age and ageing.* 2011;40(4):423-429.

49. Guralnik JM, Simonsick EM, Ferrucci L, et al. A short physical performance battery assessing lower extremity function: association with self-reported disability and prediction of mortality and nursing home admission. *Journal of gerontology.* 1994;49(2):M85-94.

50. McCaffery M, Beebe A. Pain: clinical manual for nursing practice. *CV Mosby: St Louis.* 1989.

51. Hwang SS, Chang VT, Cogswell J, Kasimis BS. Clinical relevance of fatigue levels in cancer patients at a Veterans Administration Medical Center. *Cancer.* 2002;94(9):2481-2489.

52. Kruizenga HM, Seidell JC, de Vet HC, Wierdsma NJ, van Bokhorst-de van der Schueren MA. Development and validation of a hospital screening tool for malnutrition: the short nutritional assessment questionnaire (SNAQ). *Clinical nutrition (Edinburgh, Scotland).* 2005;24(1):75-82.

53. Likert. A technique for the measurement of attitudes. In. Archives of Psychology, 140, 1–55.1932.

54. Twisk JW. *Introduction to applied biostatistics (Dutch)* 3ed. Amsterdam: Reed Business Education; 2017.

55. White IR, Royston P, Wood AM. Multiple imputation using chained equations: Issues and guidance for practice. *Stat Med.* 2011;30(4):377-399.

56. van Walraven C, Seth R, Austin PC, Laupacis A. Effect of discharge summary availability during post-discharge visits on hospital readmission. *Journal of general internal medicine.* 2002;17(3):186-192.

57. Benjamini Y. HY. Controlling the false discovery rate: a pratical and powerful approach to multiple testing. . *Journal of the Royal Statistical Society* 1995;57(1):289-300.

58. Hochberg Y. A sharper Bonferroni procedure of multiple tests of significance In. Vol 4. 75 ed. Biometrika 1988:800-802.

59. Hakkaart-van Roijen L V-d-LN, Bouwmans C, Kanters C, Tan SS. Dutch manual for costing in economic evaluations. In. Diemen National Health Care Institute 2015.

60. Lamers LM, Stalmeier PF, McDonnell J, Krabbe PF, van Busschbach JJ. [Measuring the quality of life in economic evaluations: the Dutch EQ-5D tariff]. *Nederlands tijdschrift voor geneeskunde.* 2005;149(28):1574-1578.

61. Willan AR BA. *Statistical analysis of cost-effectiveness data.* John Wiley and Sons 2006.

62. Braun V, Clarke V. Using thematic analysis in psychology. *Qualitative research in psychology.* 2006;3(2):77-101.

63. Meiland FJ, Droes RM, De Lange J, Vernooij-Dassen MJ. Development of a theoretical model for tracing facilitators and barriers in adaptive implementation of innovative practices in dementia care. *Archives of gerontology and geriatrics Supplement.* 2004(9):279-290.

For peer review only

For peer review only - http://bmjopen.bmj.com/site/about/guidelines.xhtml

64. Moore GF, Audrey S, Barker M, et al. Process evaluation of complex interventions: Medical Research Council guidance. *BMJ (Clinical research ed).* 2015;350:h1258.

65. van Seben R, Geerlings SE, Buurman BM. [10 tips for improving patient handovers from hospital to other health care settings]. *Nederlands tijdschrift voor geneeskunde.* 2017;161(0):D1031.

For peer review only

Figure 1 – Patient admission process and criteria, components of AGCH intervention and goals

(uploaded seprately as Image)

CGA= Comprehensive Geriatric Assessment[29]
GP= General Practitioner

For peer review only

For peer review only - http://bmjopen.bmj.com/site/about/guidelines.xhtml

Figure 2 Diagram of patient participation between February 1st and December 20th 2019.

(uploaded seprately as Image)

For peer review only

For peer review only - http://bmjopen.bmj.com/site/about/guidelines.xhtml

Table 1 Overview of the content and description of (outcome) measurements and timing of measurements at the Acute Community Care Hospital ('WijkKliniek')

	Description and/or instrument	H1	H2	P1	P3	P6
1. Medical and demographical data						
**Sociodemographic data**	Date of birth, age at admission, sex, level of education, living conditions, marital state	R				
**Data on admission**	Time spent at the ED, admission diagnosis, date and time of admission	R				
**Chronic conditions**	Charlson Comorbidity index[38]	R				
**Polypharmacy**	Number of drugs	R				
**Mortality**	Date of death		R	R	R	R
2. Cognitive functioning						
**Cognitive impairment**	Mini Mental State Exam (MMSE)[31]	R				
**Delirium**	Safety management system patient screening (VMS)[41]<br>Confusion Assessment Method (CAM)[39]<br>Delirium Observation Scale (DOS)[42]	N/D	N/D			
3. Psychosocial functioning and quality of life						
**Apathy**	Geriatric Depression Scale (GDS-3)[43]	N	R	R	R	R
**Social network and informal care**	Presence and frequency of informal care	R		R	R	R
**Quality of life and health status**	EQ-5D-3L[36]	R		R	R	R
4. Physical functioning						
**Identifying at-risk-patients**	ISAR-HP- Identifying Seniors at Risk score[44]	N				
**Functional status**	Activities of daily Living (ADL) modified Katz-ADL score[35]	N				
**(Im)mobility**	Using walking aid, information in KATZ-15 questions on exercise	N				
**Handgrip strength**	Jamar[48]	P				
**Gait speed**	Short Physical Performance Battery SPPB[49]	P				
**Falling**	Fall history<br>Falls in the AGCH<br>Numeric Rating scale (NRS) fear of falling[34]	N<br><br>N	R<br><br>R	R<br><br>R	R<br><br>R	R<br><br>R
**Pain**	Numeric Rating Scale (NRS) pain [50]	N	R	R	R	R
**Fatigue**	Numeric Rating Scale (NRS) fatigue [51]	N	R	R	R	R
**Nutrition**	Short Nutritional Assessment Questionnaire (SNAQ- Score)[52]	N				
5. Healthcare utilization and satisfaction with care						
**Medical care during admission**	Diagnostics performed in the AGCH<br>Readmission to university hospital<br>Length of stay at the AGCH		R			
**Hospital readmission**	Readmission rate to the hospital or AGCH		R	R	R	R

For peer review only

For peer review only - http://bmjopen.bmj.com/site/about/guidelines.xhtml

**Health care utilization**	Home care, medical specialist care, temporary institutional care, primary care.	R		R	R	R
**Satisfaction with Care**	8 question questionnaire[53]		R	(R) *		

H1= at admission, H2= at discharge, P1= one month after discharge, P3 = three months after discharge, P6 = six months after discharge. N=nurse Geriatric Community Care Hospital P= physiotherapist D= Doctor/attending physician R= researcher/research nurse *in case assessment was missed at H2

For peer review only

For peer review only - http://bmjopen.bmj.com/site/about/guidelines.xhtml

Table 2 Baseline characteristics of study participants

**Variable**	**N=212**
**Age in years,** mean (SD)	81.8 (8.4)
**Male,** N(%)	101 (47.6)
**Living arrangements before admission,** N (%)	
Independent	172 (81.1)
Assisted living/ senior residence	31 (14.6)
Nursing home/other	9 (4.2)
**Marital status,** N **(%)**	
Widow/widower	94 (44.5)
Married or living together	71 (33.6)
Single or divorced	46 (21.8)
**Education,** N(%)	
Primary school	36 (18.7)
Elementary technical/domestic science school	41 (21.2)
Secondary vocational education	65 (33.7)
Higher level high school/third-level education	51 (26.4)
**Born in the Netherlands,** N (%)	158 (76.0)
**ADL-score upon admission (KATZ-6[a]),** median (IQR)	3.0 (1.0-5.0)
**MMSE score[b],** mean (SD)	23.7 (4.7)
**Polypharmacy[c],** N(%)	159 (75.0)
**Hospitalization in past 6 months,** N (%)	61 (31.1)
**Charlson Comorbidity Index[d]** (mean, SD)	2.8 (2.0)
**Primary admission diagnosis,** N (%)	
Infectious diseases	60 (28.3)
Respiratory (including pneumonia)	54 (25.5)
Gastrointestinal	9 (4.2)
Cardiac	20 (9.4)
Neurology	16 (7.5)
Other (e.g. falls, delirium, sudden unexplained functional decline)	53 (30.2)

SD, standard deviation; IQR, interquartile range
[a]Score ranging from 0-6,with a higher score indicating more dependence in activities of daily living[35]
[b]Score ranging from 0-30, with a score of ≤23 indicating possible cognitive impairment [31]
[c]Use of 5 drugs or more
[d]Range of 0-31, with a higher score indicating more severe comorbidity[38]

For peer review only - http://bmjopen.bmj.com/site/about/guidelines.xhtml

For peer review only

For peer review only - http://bmjopen.bmj.com/site/about/guidelines.xhtml

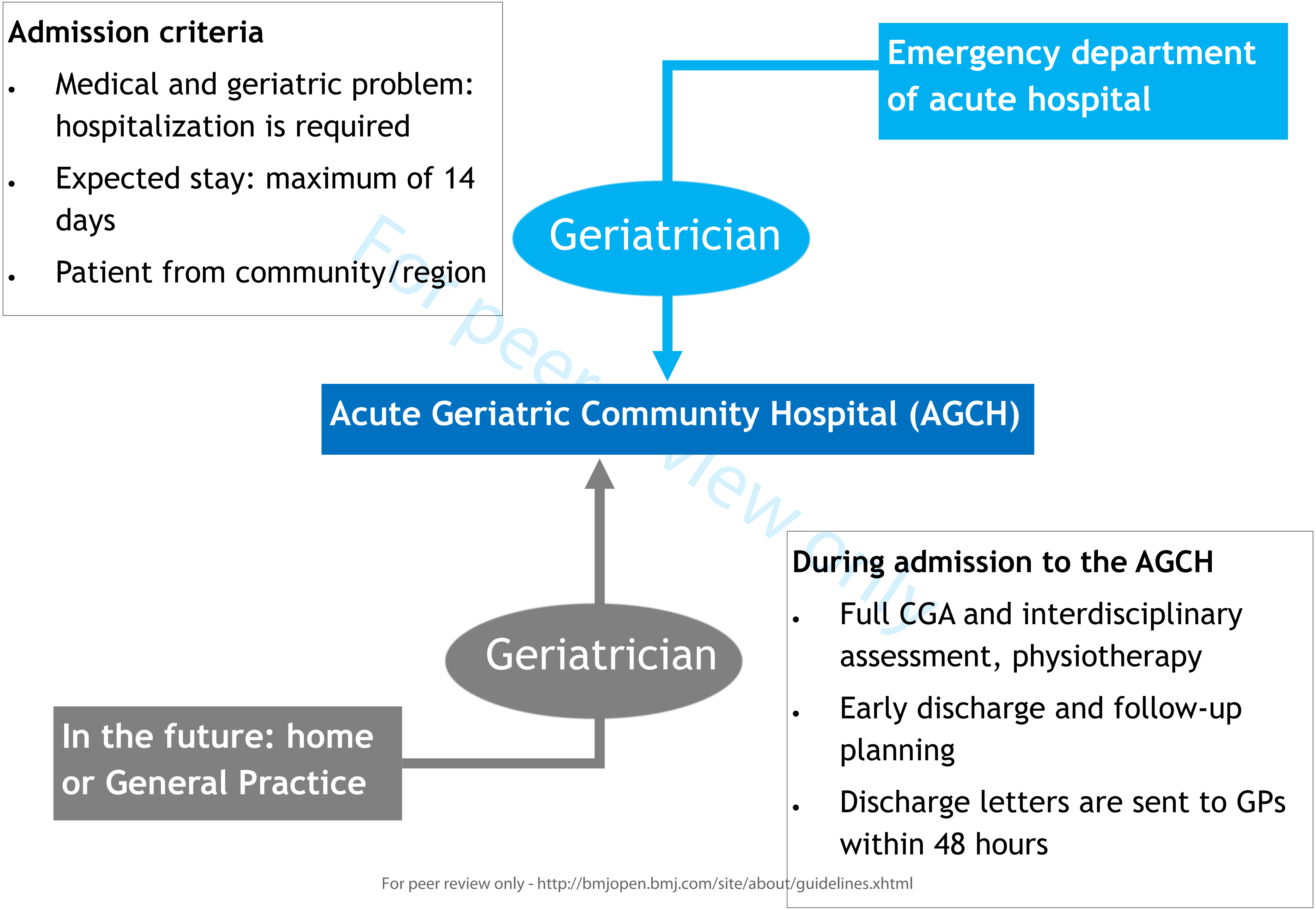
Admission criteria
Medical and geriatric problem: hospitalization is required
Expected stay: maximum of 14 days
Patient from community/region
Emergency department of acute hospital
Geriatrician
Acute Geriatric Community Hospital (AGCH)
Geriatrician
In the future: home or General Practice
During admission to the AGCH
Full CGA and interdisciplinary assessment, physiotherapy
Early discharge and follow-up planning
Discharge letters are sent to GPs within 48 hours

Figure 2 Diagram of patient participation between February 1st and December 20th 2019.

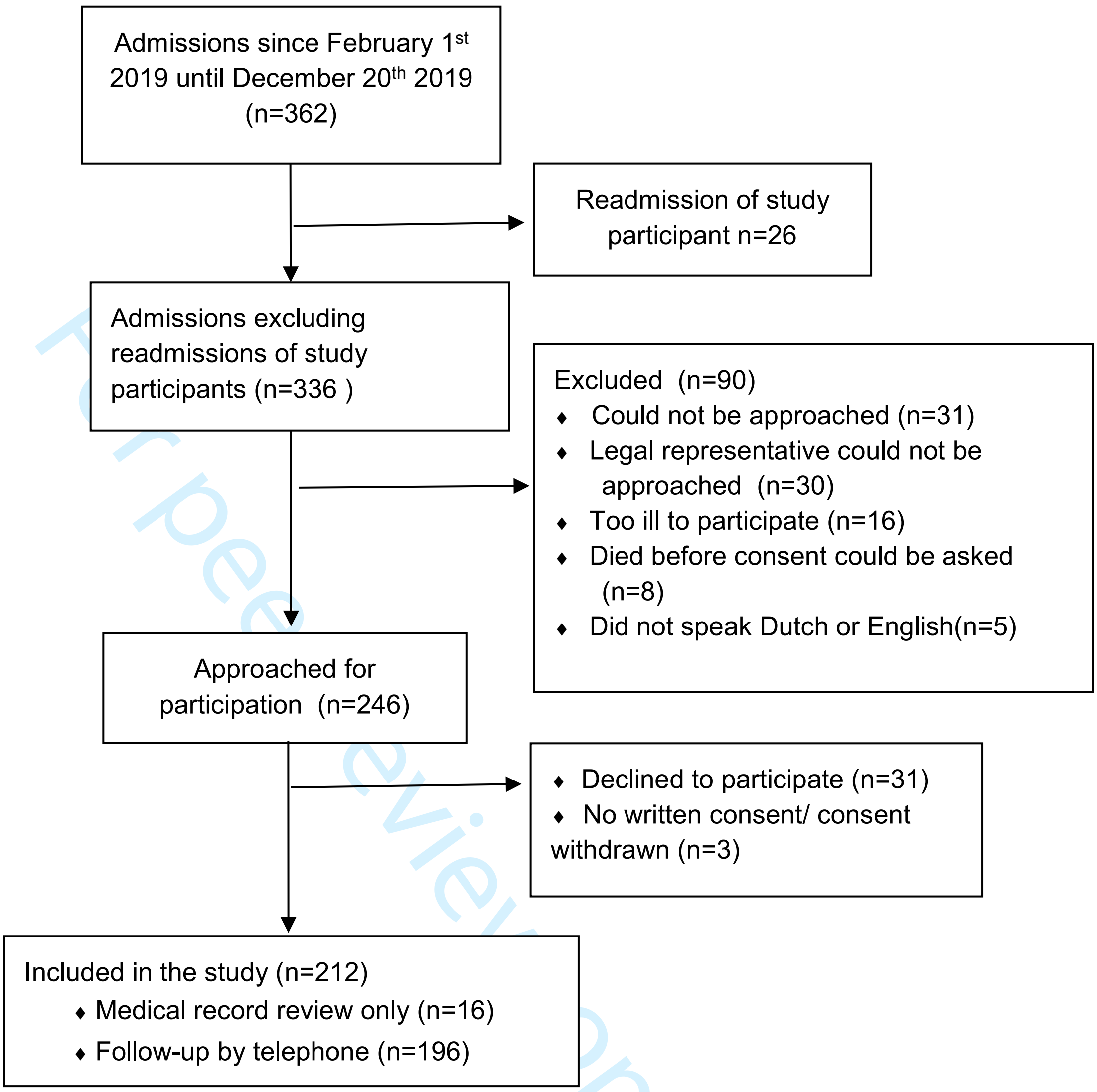

Appendix 1 STROBE statement checklist

STROBE Statement—Checklist of items that should be included in reports of ***cohort studies***

	Item No	Recommendation	Item found on page
**Title and abstract**	1	(*a*) Indicate the study's design with a commonly used term in the title or the abstract	1 and 3
		(*b*) Provide in the abstract an informative and balanced summary of what was done and what was found	Page 3
**Introduction**			
Background/rationale	2	Explain the scientific background and rationale for the investigation being reported	Page 5-7
Objectives	3	State specific objectives, including any prespecified hypotheses	Page 7
**Methods**			
Study design	4	Present key elements of study design early in the paper	Page 8 -9
Setting	5	Describe the setting, locations, and relevant dates, including periods of recruitment, exposure, follow-up, and data collection	Page 7-15
Participants	6	(*a*) Give the eligibility criteria, and the sources and methods of selection of participants. Describe methods of follow-up	Page 7-10 and 11
		(*b*) For matched studies, give matching criteria and number of exposed and unexposed	Page 9 and 10
Variables	7	Clearly define all outcomes, exposures, predictors, potential confounders, and effect modifiers. Give diagnostic criteria, if applicable	Page 11 – 15
Data sources/ measurement	8*	For each variable of interest, give sources of data and details of methods of assessment (measurement). Describe comparability of assessment methods if there is more than one group	Page 11-15
Bias	9	Describe any efforts to address potential sources of bias	Page 16
Study size	10	Explain how the study size was arrived at	Page 15
Quantitative variables	11	Explain how quantitative variables were handled in the analyses. If applicable, describe which groupings were chosen and why	Page 16
Statistical methods	12	(*a*) Describe all statistical methods, including those used to control for confounding	Page 16

For peer review only

		(*b*) Describe any methods used to examine subgroups and interactions	n/a
		(*c*) Explain how missing data were addressed	Page 16
		(*d*) If applicable, explain how loss to follow-up was addressed	Page 16
		(*e*) Describe any sensitivity analyses	n/a
**Results**			
Participants	13*	(a) Report numbers of individuals at each stage of study—eg numbers potentially eligible, examined for eligibility, confirmed eligible, included in the study, completing follow-up, and analysed	Page 18 and 26 (figure 2)
		(b) Give reasons for non-participation at each stage	Page 18 and 26 (figure 2)
		(c) Consider use of a flow diagram	Page 26, figure 2
Descriptive data	14*	(a) Give characteristics of study participants (eg demographic, clinical, social) and information on exposures and potential confounders	Page 18 and 29
		(b) Indicate number of participants with missing data for each variable of interest	n/a
		(c) Summarise follow-up time (eg, average and total amount)	n/a
Outcome data	15*	Report numbers of outcome events or summary measures over time	n/a
Main results	16	(*a*) Give unadjusted estimates and, if applicable, confounder-adjusted estimates and their precision (eg, 95% confidence interval). Make clear which confounders were adjusted for and why they were included	n/a
		(*b*) Report category boundaries when continuous variables were categorized	Page 29
		(*c*) If relevant, consider translating estimates of relative risk into absolute risk for a meaningful time period	n/a
Other analyses	17	Report other analyses done—eg analyses of subgroups and interactions, and sensitivity analyses	n/a
**Discussion**			
Key results	18	Summarise key results with reference to study objectives	Page 19

For peer review only

Limitations	19	Discuss limitations of the study, taking into account sources of potential bias or imprecision. Discuss both direction and magnitude of any potential bias	Page 19
Interpretation	20	Give a cautious overall interpretation of results considering objectives, limitations, multiplicity of analyses, results from similar studies, and other relevant evidence	Page 19
Generalisability	21	Discuss the generalisability (external validity) of the study results	n/a
**Other information**			
Funding	22	Give the source of funding and the role of the funders for the present study and, if applicable, for the original study on which the present article is based	Page 20

*Give information separately for exposed and unexposed groups.

Note: An Explanation and Elaboration article discusses each checklist item and gives methodological background and published examples of transparent reporting. The STROBE checklist is best used in conjunction with this article (freely available on the Web sites of PLoS Medicine at http://www.plosmedicine.org/, Annals of Internal Medicine at http://www.annals.org/, and Epidemiology at http://www.epidem.com/). Information on the STROBE Initiative is available at http://www.strobe-statement.org.

For peer review only

BMJ Open

Investigating the effectiveness of care delivery at an acute geriatric community hospital for older adults in the Netherlands: a protocol for a prospective controlled observational study

Journal:	*BMJ Open*
Manuscript ID	bmjopen-2019-033802.R2
Article Type:	Protocol
Date Submitted by the Author:	21-Feb-2020
Complete List of Authors:	Ribbink, Marthe; Amsterdam University Medical Centres, Department of Internal Medicine, Section of Geriatric Medicine<br>Macneil-Vroomen , Janet; Amsterdam University Medical Centres, Department of Internal Medicine, Section of Geriatric Medicine ; Yale School of Medicine, Department of Internal Medicine, Section of Geriatrics<br>van Seben, Rosanne; Amsterdam University Medical Centres, Department of Internal Medicine, Section of Geriatric Medicine<br>Oudejans , Irène; Amsterdam University Medical Centres, Department of Internal Medicine, Section of Geriatric Medicine<br>Buurman, Bianca; Amsterdam University Medical Centres, Department of Internal Medicine, Section of Geriatric Medicine ; Amsterdam University of Applied Sciences, ACHIEVE- Centre of Applied Research, Faculty of Health
**Primary Subject Heading**:	Geriatric medicine
Secondary Subject Heading:	Health services research, Public health
Keywords:	GERIATRIC MEDICINE, OLDER ADULTS, COMMUNITY HOSPITAL, INTERMEDIATE CARE FACILITIES, READMISSIONS, FUNCTIONAL DECLINE

SCHOLARONE™
Manuscripts

I, the Submitting Author has the right to grant and does grant on behalf of all authors of the Work (as defined in the below author licence), an exclusive licence and/or a non-exclusive licence for contributions from authors who are: i) UK Crown employees; ii) where BMJ has agreed a CC-BY licence shall apply, and/or iii) in accordance with the terms applicable for US Federal Government officers or employees acting as part of their official duties; on a worldwide, perpetual, irrevocable, royalty-free basis to BMJ Publishing Group Ltd ("BMJ") its licensees and where the relevant Journal is co-owned by BMJ to the co-owners of the Journal, to publish the Work in this journal and any other BMJ products and to exploit all rights, as set out in our licence.

The Submitting Author accepts and understands that any supply made under these terms is made by BMJ to the Submitting Author unless you are acting as an employee on behalf of your employer or a postgraduate student of an affiliated institution which is paying any applicable article publishing charge ("APC") for Open Access articles. Where the Submitting Author wishes to make the Work available on an Open Access basis (and intends to pay the relevant APC), the terms of reuse of such Open Access shall be governed by a Creative Commons licence – details of these licences and which Creative Commons licence will apply to this Work are set out in our licence referred to above.

Other than as permitted in any relevant BMJ Author's Self Archiving Policies, I confirm this Work has not been accepted for publication elsewhere, is not being considered for publication elsewhere and does not duplicate material already published. I confirm all authors consent to publication of this Work and authorise the granting of this licence.

For peer review only

Investigating the effectiveness of care delivery at an acute geriatric community hospital for older adults in the Netherlands: a protocol for a prospective controlled observational study

Marthe E. Ribbink MD[1], Janet L. MacNeil Vroomen PhD[1,2], Rosanne van Seben PhD[1], Irène Oudejans MD, PhD[1], Bianca M. Buurman RN, PhD[1,3] on behalf of the AGCH study group.

[1] Amsterdam University Medical Centre, University of Amsterdam, Department of Internal Medicine, Section of Geriatric Medicine, Amsterdam Public Health Research Institute, Meibergdreef 9, Amsterdam, The Netherlands

[2] Section of Geriatrics, Department of Internal Medicine, School of Medicine, Yale School of Medicine, The United States

[3] ACHIEVE – Centre of Applied Research, Faculty of Health, Amsterdam University of Applied Sciences, Amsterdam, The Netherlands

Word count, references and tables

Abstract: 264

Total word count: 4600

Number of references: 65

Number of tables/figures: 4, 2 tables and 2 figures

Supplementary material: STROBE checklist and 1 supplementary table

Authors' email addresses

M.E. Ribbink, m.e.ribbink@amsterdamumc.nl

J.L MacNeil Vroomen j.l.macneil-vroomen@amsterdamumc.nl

R. van Seben rosanne.vanseben@ecorys.com

I. Oudejans i.oudejans@amc.uva.nl

B.M. Buurman b.m.vanes@amsterdamumc.nl

Corresponding author

M.E. Ribbink

Amsterdam UMC, University of Amsterdam, Department of Internal Medicine, Section of Geriatric Medicine, D3-335

PO Box 22600, 1100 DD Amsterdam, The Netherlands

Tel: 0031-20 5661647, Email: m.e.ribbink@amsterdamumc.nl

For peer review only

For peer review only - http://bmjopen.bmj.com/site/about/guidelines.xhtml

Keywords: geriatric medicine, older adults**,** community hospital, intermediate care facilities, readmissions, functional decline.

For peer review only - http://bmjopen.bmj.com/site/about/guidelines.xhtml

Abstract

Introduction: Hospital admission in older adults with multiple chronic conditions is associated with unwanted outcomes like readmission, institutionalization, functional decline, and mortality. Providing acute care in the community and integrating effective components of care models might lead to a reduction in negative outcomes. Recently, the first geriatrician-led Acute Geriatric Community Hospital (AGCH) was introduced in the Netherlands. Care at the AGCH is focused on the treatment of acute diseases, comprehensive geriatric assessment, setting patient-led goals, early rehabilitation and streamlined transitions of care.

Methods and analysis: This prospective cohort study will investigate the effectiveness of care delivery at the AGCH on patient outcomes by comparing AGCH patients to two historic cohorts of hospitalized patients. Propensity score matching will correct for potential population differences. The primary outcome is the three-month unplanned readmission rate. Secondary outcomes include functional decline, institutionalization, healthcare utilization, occurrence of delirium or falls, health-related quality of life, mortality and patient satisfaction. Measurements will be conducted at admission, discharge and one, three and six months after discharge. Furthermore, an economic evaluation and qualitative process evaluation to assess facilitators and barriers to implementation are planned.

Ethics and dissemination: The study will be conducted according to the Declaration of Helsinki. The Medical Ethics Research Committee (METC) confirmed that the Medical Research Involving Human Subjects Act did not apply to this research project and official approval was not required. The findings of this study will be disseminated through public lectures, scientific conferences and journal publications. Furthermore, the findings of this study will aid in the implementation and financing of this concept (inter)nationally.

Trial Registration Number NL7896; pre-results

For peer review only - http://bmjopen.bmj.com/site/about/guidelines.xhtml

Strengths and limitations of this study:

- This study will be the first to evaluate an acute geriatric community hospital in the Netherlands on both patient-reported and economic outcomes.
- Patients, informal caregivers and professionals were involved in the design and implementation of the Acute Geriatric Community Hospital (AGCH).
- A process evaluation is planned to describe the experience of various stakeholders with this new concept and reveal barriers and facilitators to its implementation.
- A limitation of this study is the use of two historic cohorts as the control population, which may result in baseline differences between the control and intervention population.

For peer review only

For peer review only - http://bmjopen.bmj.com/site/about/guidelines.xhtml

Introduction

Background

Throughout the western world, there is an increase in older adults requiring acute care. Inpatient services are mostly consumed by those over the age of 65.[1,2] The Netherlands, like many other countries, recently (2015) implemented stay-at-home policies leading to an increase in frail older persons living longer in the community.[3] These reforms juxtaposed with an increased ageing population contribute to increased acute care utilization.[4] There has been a 19% increase in emergency department (ED) visits by Dutch older adults based on data from 2015 versus 2017.[5,6]

Many older adults come to the hospital with complex and atypical health problems.[5,7] When older persons are subsequently hospitalized, health outcomes are known to be poor,[8] particularly in patients with geriatric syndromes such as cognitive impairment or mobility impairment.[9,10] For example, previous research showed that 30% of older persons gained new disabilities and 20% were readmitted within 30 days postdischarge.[11,12] Hospitalization itself may contribute to these poor outcomes, as hospitalized older adults often have reduced mobility because they are bedbound for approximately 20 hours a day.[13,14] Low physical activity, in combination with poor nourishment and increased caloric demand due to acute illness, can lead to the loss of muscle mass and may contribute to the development of new disabilities, particularly in frail patients.[15,16] Together with the noise in a hospital environment and the different personnel rotating between patient rooms, this contributes to sensory overstimulation and sleep deprivation, which may lead to confusion and the occurrence of delirium.[17,18,19] Not only is the patient affected during hospitalization but the informal caregivers also find hospital admissions stressful.[20] Furthermore, previous research shows that a lack of discharge planning in the hospital can result in patients' care needs being unmet.[21] Hospital care as usual compared to discharge planning and follow-up show a higher rate of early readmissions.[22] Readmissions can further affect patients' recovery and increase healthcare costs.[23]

For peer review only - http://bmjopen.bmj.com/site/about/guidelines.xhtml

134 The complex medical needs of older persons, combined with their more dependent social situation, requires care delivery that offers guidance and support for realistic health and life goals.[24] Perhaps a 'gap' exists between what care can be provided in an acute care hospital versus what can be provided in the community (primary care). Acute hospital care is secondary care with a focus on medical treatment and diagnostics, whilst primary care focuses on rehabilitation, nursing care and wellbeing.

140 Several alternative strategies to hospital admission and (nurse-led) intermediate care have been developed in the past as a substitute to conventional hospitalization.[25] Examples include (nurse-led) intermediate care and subacute geriatric care units, which are low-tech but with geriatric expertise.[26,27] In general, these types of care have comparable outcomes to hospital care as usual. Moreover, nurse-led care in the United States, observation units and hospital at home care all show a cost reduction compared to care as usual.[25,26] Until recently, the Netherlands had limited alternatives to hospitalization for older persons who required acute care. Therefore, our research group sought to create an acute care alternative and opened the Acute Geriatric Community Care Hospital (AGCH) in July 2018, partnering with an academic hospital (Amsterdam UMC, location AMC), an insurance company (Zilveren Kruis) and a home care and nursing home agency (Cordaan). This acute geriatric care unit, which is based within an intermediate care facility, provides an alternative to conventional hospitalization and delivers acute care closer to home.

152 The AGCH delivers acute care that is focused on early mobilization and rehabilitation. Older persons with common medical problems (such as urinary tract infections, pneumonia or heart failure) and geriatric syndromes requiring hospital admission can be admitted to the AGCH. The AGCH provides a form of *intermediate* care between primary and secondary care. In the Netherlands, primary care includes general practice, community nursing and (temporary) admission to a nursing home. Secondary care includes specialist medical care and hospital admission. The care at the AGCH is supervised by a geriatrician and provided by nurses trained in geriatric care who have experience as

For peer review only - http://bmjopen.bmj.com/site/about/guidelines.xhtml

either a hospital or community nurse. The single rooms are designed to accommodate respite for the informal caregivers. This concept of care is new to the Netherlands, and to our knowledge, there is only one comparable example in Europe: a "subacute care unit" in intermediate care, which has been implemented in Spain.[27]

Our hypothesis is that with the provision of integrated medical and nursing care close to home, the AGCH is better suited to the needs of older adults with multiple chronic conditions and will lead to better patient health outcomes and reduced post-acute care costs. Therefore, this study is designed to compare care provided for older patients in the AGCH versus care provided in a hospital setting. Specifically, we aim to:

- Evaluate the 90-day readmission rate of patients acutely admitted to the AGCH compared to a traditional hospital (usual care). Secondary outcomes include functional decline, institutionalization, healthcare utilization, the occurrence of geriatric syndromes such as delirium, health-related quality of life, mortality, and patient satisfaction;
- Assess the cost-effectiveness of the AGCH versus usual care by performing an economic evaluation from a health care provider and societal perspective;
- Conduct a process evaluation using interviews with key stakeholders to identify facilitators and barriers to the implementation of the AGCH.

Methods

Setting

The AGCH opened in July 2018. It serves the south-eastern part of Amsterdam and its surrounding areas (an area with approximately 147 500 inhabitants).[28] The AGCH is a 23-bed facility within a skilled nursing facility. The hospital has 24-hour geriatric and nursing assistance. Physiotherapy and routine laboratory testing are available during the workweek and simple X-ray is available once a week. The population that is eligible for admission to the AGCH are patients with a combination of an

For peer review only - http://bmjopen.bmj.com/site/about/guidelines.xhtml

acute medical problem requiring hospitalization (e.g., pneumonia, exacerbation of heart failure or a urinary tract infection), and a geriatric condition (e.g., delirium, cognitive impairment, falls, or functional impairment). Additionally, patients have to be haemodynamically stable and should not require complex diagnostic testing. In general, patients will not be admitted if they have the following exclusion criteria: 1) require care that can only be provided at an intensive care unit, 2) require surgery, 3) require urgent treatments or diagnostic tests that can only be provided in-hospital (e.g., endoscopy, interventional radiology), 4) do not need hospital care but require transfer to a skilled nursing facility and 5) live in another region of the Netherlands.

Patients are directly admitted to the AGCH from the ED of the Amsterdam UMC-location Academic Medical Centre (AMC) in Amsterdam, which is a 1000-bed academic hospital with approximately 30 000 ED visits yearly. After the on-call geriatrician has assessed whether the patient is eligible for AGCH admission and the patient or representative has agreed to admission, the patient is transferred to the AGCH by ambulance. Since October 2019, patients can also be transferred from the EDs of other hospitals in Amsterdam. In the future, we plan to admit patients from home or general practice offices. Patients are admitted between 8.00 am and 11.00 pm, seven days a week. At admission, a Comprehensive Geriatric Assessment (CGA) is conducted.[29] The CGA gives an overview of all medical, functional, psychological and social problems. The CGA is discussed during multidisciplinary team meetings and used to formulate a care plan for each patient. For an overview of the admission process, the admission criteria and the components of this intervention, see figure 1.

Study design

This study is a prospective, observational, cohort study with two historical control groups to evaluate the clinical and economic effects of the AGCH. The STROBE statement was used in preparing the study protocol (appendix 1).[30] Participants will be compared to hospital controls. The participants are recruited into the study and are assessed at admission, discharge, and one, three and six months after discharge. Recruitment for this study started in February 2019. We plan to recruit for 18 to 24

For peer review only - http://bmjopen.bmj.com/site/about/guidelines.xhtml

months. The first three months of data collection consisted of a piloting phase to assess the feasibility of data collection and follow-up. In addition, a qualitative process evaluation on the facilitators and barriers to the implementation of the AGCH and patient experience will be conducted.

Participants

Patients admitted to the AGCH are eligible for inclusion in the study. However, patients are excluded from the study if: 1) the attending physician judges that the patient is too ill to participate, e.g., the patient is terminally ill, 2) the patient or legal representative does not consent to participate, or 3) the patient or legal representative does not speak or understand Dutch or English. In the case of cognitively impaired or delirious patients, patients can only be included if a legal representative consents to participation and acts as healthcare proxy. Cognitive functioning is assessed by the attending physician and confirmed by the researcher by conducting a Mini-Mental State Examination (MMSE).[31] An MMSE score of 15 or less indicates severe cognitive impairment, in which the approval of a legal representative will be sought.

Historical control groups

We selected two completed cohort studies that were conducted by our research group as historical control groups. We expect that the patients from these cohorts have similar admission diagnoses as those who can be admitted to the AGCH, namely, diagnoses that are ambulatory care sensitive conditions such as infections and exacerbations of COPD or heart failure. Patients in these two cohorts were admitted to internal medicine, cardiology, pulmonology and geriatrics departments. These departments admit patients with diagnoses similar to those that can be admitted to the AGCH. In addition, we have selected these cohorts as control groups as the patients come from the same area as the studied population admitted to the AGCH, that is, the greater Amsterdam area. The first

For peer review only

control group from the Transitional Care Bridge Study consists of 674 patients who were recruited between September 2010 and March 2014.[32] Participants were patients of 65 years and older hospitalized for at least 48 hours. Proxy consent was provided for participants suffering from severe cognitive impairment (MMSE ≤15). They participated in a negative randomized controlled trial that assessed the effectiveness of a nurse-led transitional care programme in preventing functional decline.[32] The second control group from Hospital-ADL study consists of 401 patients who were recruited between October 2015 and June 2017.[10] These participants were enrolled in a prospective cohort studying the trajectory of functional decline in older hospitalized adults. Participants were aged 70 years and older and were hospitalized for at least 48 hours. Patients suffering from severe cognitive impairment (MMSE ≤15) and delirium were excluded from participation. For the detailed methodology and inclusion criteria of the two control cohorts, please refer to the study protocols and papers of these studies.[10,32-34]

Patient and public involvement

Older persons living in Amsterdam were involved in the design of the AGCH concept. No patients were involved in the design of this study.

Outcomes

The primary outcome measure is the 3-month unplanned readmission rate to the AGCH or hospital. Secondary outcomes measured at one, three and six months will include:

1) Activities of daily living (ADL)-functioning, as defined by the Katz-ADL scale.[35]
2) Healthcare utilization, including institutionalization in a long-term care facility.
3) Occurrence of delirium and/or falls.
4) Health-related quality of life (HRQOL).[36]
5) All-cause mortality.
6) Satisfaction of the patients and primary caregivers with the provided care.

For peer review only - http://bmjopen.bmj.com/site/about/guidelines.xhtml

Data collection

Eligible patients and/or legal representatives will be contacted and informed about the study procedures after which written informed consent is obtained. Inclusion and interviewing of patients is conducted by an onsite researcher. Routine data on functioning and risk assessments are collected by a trained registered nurse and physiotherapist as part of the CGA for each patient.[37] Table 1 gives an overview of measurement of the primary and secondary outcomes over time. These measurements were chosen based on the assessments and data collected from the two historic control groups. The supplementary table provides an overview of the content and timing of measurements in the AGCH-group compared to the two historic control groups. Measurements during admission are at H1, which is within 48 hours after admission, and H2, which is within 48 hours before discharge. Follow-up is completed by telephone at one, three and six months after discharge (P1, P3 and P6).

Data collection includes:

1. Medical and demographical data

Sociodemographic data. These will include age, gender, highest level of education, ethnicity, marital status and living arrangement.

Time spent at the ED, admission diagnosis, and date and time of admission.

Chronic conditions. The number and severity of chronic conditions will be assessed using the Charlson Comorbidity Index.[38] This index is commonly used to indicate the risk of mortality; each condition is scored 1, 2, 3 or 6 points, with a higher total number of points indicating a greater risk of death.

Polypharmacy. Polypharmacy will be assessed by counting the number of individual drugs that are chronically prescribed to a participant, in which a number of 5 or more drugs is considered polypharmacy.

For peer review only - http://bmjopen.bmj.com/site/about/guidelines.xhtml

Mortality. This will be assessed during follow-up, either from the patients' electronic files or from general practice registries.

2. Cognitive functioning.

Cognitive impairment. This is assessed by reviewing the score of the MMSE that is performed within 48 hours of admission. The MMSE includes 23 items (total score 0-30) that screen for cognitive impairment. A score of 23 or less is defined as possible cognitive impairment.[31] When a patient is delirious upon inclusion, the MMSE is not conducted.

Delirium. The Confusion Assessment Method (CAM), 4 item short version, is used to assess the presence and duration of delirium.[39] The CAM is widely used by physicians and nurse practitioners to diagnose delirium (sensitivity of 53-90% and specificity of 84-100%).[40] The CAM is filled out within 24 hours of admission. Moreover, the risk on developing delirium is assessed using the Dutch Safety Management Programme (*Veiligheidsmanagementsysteem (VMS)*) criteria for risk of delirium.[41] Nurse practitioners will score the CAM daily from day one till day three of admission; if there are signs of possible delirium at day 3, these measurements are continued until the symptoms are resolved. In addition, during the first three days of admission, the Delirium Observation Screening Scale (DOSS) is scored during each nursing shift and is continued when there is a clinical suspicion of delirium.[42]

3. Psychosocial functioning and quality of life

Apathy. We use three items of the *Geriatric Depression Scale* (GDS-15) to assess apathy (sensitivity of 69% and specificity of 85 %). These items include the following questions: 1) 'Do you prefer to stay at home, rather than going out and doing new things', 2) 'Have you dropped many of your activities and interests?', and 3) 'Do you feel full of energy'. A score of >2 points is classified as 'apathy present'. [43]

For peer review only

Social network and informal care. Participants are asked if they receive informal care, how many hours a week, what type of care (housekeeping and/or personal care) and from which persons (partners, children, other family members or neighbours/volunteers).

Health-related quality of life. This will be measured by the EuroQoL-5D (EQ-5D). The EQ-5D is a broadly used and validated instrument for measuring generic health-related quality of life.[36]

4. Physical functioning

Risk of functional decline. Patients are assessed for risk of functional decline using the Identification of Seniors at Risk- Hospitalized Patients (ISAR-HP) tool; scores of two and up indicate an increased risk for functional decline.[44]

Functioning level. The 15-item modified Katz-ADL score is used to measure ADL functioning. This includes statements about independence in performing basic activities of daily living (ADL) and in instrumental activities of daily living (IADL).[45,46] We measure the Katz-ADL both currently (at admission), as well as two weeks before admission, reflecting pre-morbid level of functioning. The Katz-ADL is also measured during follow-up.

(Im)mobility. Mobility is assessed by reviewing three questions that are in the admission assessment regarding: 1) the use of a walking aid, 2) being able to walk outside of the house for five minutes (two weeks before and currently) and 3) the performance and frequency of physical activity.[47]

Handgrip strength. Measure muscle weakness is measured by physiotherapists in all admitted patients using the maximum handgrip strength (Jamar). [48]

Gait speed. Gait speed is measured as part of the Short Physical Performance Battery (SPBB), which is part of the physiotherapists' admission assessment.[49]

For peer review only - http://bmjopen.bmj.com/site/about/guidelines.xhtml

Falls. Fall history is assessed by asking about the number of falls in the past six months.[41] During the discharge assessment, the occurrence of falls in the AGCH and the consequences of falls (indication for prolonged stay, diagnostics or injury) are recorded.

Fear of falling. The Numeric Rating Scale (NRS, score 0-10) is used to assess the fear of falling; 0 indicates no fear of falling, and 10 indicates the greatest fear of falling possible.[34]

Pain. The standard clinical measure for pain is the NRS, ranging from 0 to 10, in which a score of 0 represents no pain and 10 represents the worst possible pain.[50]

Fatigue. A NRS from 0-10 is used, with 0 indicating no fatigue and 10 indicating the greatest fatigue ever felt by the participant.[51]

Sleep. Participants are asked if they have had difficulties with sleeping in the past month and whether participants have used sleep medication.

Nutrition. We will use the Short Nutritional Assessment Questionnaire (SNAQ) to identify patients with malnourishment. The SNAQ consists of three questions concerning weight loss, appetite and drink/tube nutrition, resulting in a score ranging from 0 to 5. Scores of 0 and 1 are defined as 'no malnutrition', 2 as 'moderate malnutrition' and 3 or more as 'severe malnutrition'.[52]

5. Healthcare utilization and satisfaction with care

Medical care during admission and the process of discharge. The following items are collected from patients' electronic health records: the diagnostics performed in the AGCH, revisits to the hospital, admissions to the hospital, length of stay at the AGCH, discharge destination and time needed to send medical handovers to the general practitioner.

Hospital readmission. This outcome will be assessed during follow-up. Follow-up will consist of three telephone interviews at one, three and six months after discharge. Readmission will be both assessed during the follow-up interviews and by checking care data from an aggregated database of expense

For peer review only - http://bmjopen.bmj.com/site/about/guidelines.xhtml

claims from various healthcare insurers. Data that will be collected are as follows: number of readmissions, total days of readmission, reasons for readmission and whether the readmission was planned or unplanned.

Emergency department (ED) visits. ED visits will be assessed during follow-up and checked in the insurance data. We will record the number of separate ED visits.

Outpatient hospital visits. We will ask patients if there have been any outpatient visits in the past month(s), and if so, how many.

Consultations by general practitioners. We will ask patients if, and how many times, they have consulted with their general practitioner (both during the day and during out-of-office hours).

Consultations by physiotherapists or dieticians. We will ask patients if, and how many times, they have consulted with a physiotherapist or dietician in the past month(s).

Home care. This includes questions on the frequency of home care, including housekeeping, personal care and nursing care. We will also include hours of informal care provided by family members or friends.

Temporary admission to a nursing home. This includes days of (temporary) admission to a skilled nursing facility or rehabilitation facility.

Permanent institutionalization. This concerns long-term admission to a skilled nursing facility and the date of admission to this facility.

Patient satisfaction with care. Patients or informal caregivers are asked to fill out an 8-question questionnaire regarding their satisfaction with the care that they received. Questions are answered on a 5-point Likert scale.[53]

For peer review only - http://bmjopen.bmj.com/site/about/guidelines.xhtml

For peer review only

Sample size calculation

In the Hospital-ADL study, 34% of participants experienced a readmission at 90 days.[34] Assuming that 26% of patients admitted to the AGCH will experience a 90-day readmission, data from 515 patients at the AGCH will yield 80% power to detect an absolute difference of 8% in the readmission rate (which is a 25% reduction in the relative risk) using a two-sided test with an alpha of 0.05.[54] As we expect 10% loss to follow-up, we aim to include a total of 567 (= 515*1.10) patients from the AGCH.

Planned statistical analyses

The complete participant flow diagram will show a summary of admissions and study recruitment at the AGCH and will provide study discontinuation rates at one, three and six months follow-up.[30] We will describe the demographic, clinical and prognostic characteristics of the study participants at baseline. The number of participants with missing data will be collected and described alongside our variables to check for the pattern of missingness. Inversely weighted propensity scores will be used to control for any imbalances between the treatment groups.[55] Propensity scores will be calculated using generalized booted methods. Balance and overlap of propensity score distribution will be assessed. Propensity score weights for the estimation of the average treatment effect will be created using all covariates where groups differed at baseline or that were associated with the 90-day readmission rate. As this is a repeated measures design, we will assume equal weighting for all measurements.[56]

All hypotheses will be tested using a two-tailed significance level of 0.05. All secondary outcomes will be adjusted for multiple testing using a Hochberg method.[57,58] Descriptive analyses will be performed to examine the participants' characteristics. Differences in changes over time in outcomes will be compared between groups using multilevel models. All models will include a main effect of treatment group, a linear term for time and an interaction between time and treatment group. Models will be checked with residual and appropriate goodness-of-fit statistics.

For peer review only - http://bmjopen.bmj.com/site/about/guidelines.xhtml

For peer review only

Economic evaluation

A healthcare and societal perspective is planned for the economic evaluation. The evaluation from the healthcare perspective will only include direct medical costs accrued in the six months after the admission to the AGCH. Direct medical costs will only include costs that are funded through the Dutch healthcare system. The evaluation from a societal perspective will include an estimation of the costs of informal care. Costs will be based on the reference prices found in the Dutch Manual for Costing studies and will be set for the final year of data collection (2020 or 2021). According to this guideline, costs will be discounted at 4% and quality adjusted life years (QALY) will be discounted at 1.5%. [59] Propensity scores will also be used in the economic evaluation. Missing data will be imputed using multiple imputation chained equations, if necessary, for the cost and effect data. We plan to use generalized linear regression models with a gamma distribution and an identity link to account for the right skew of the cost data. A generalized linear regression model will be used to estimate the incremental effect in QALY adjusted for baseline utility estimates with a Gaussian distribution and identify link.[60] Incremental cost-effectiveness ratios will be calculated using the pooled cost and effect estimates. Bootstrapped cost-effect pairs will be plotted on a cost-effectiveness plane and used to estimate cost-effectiveness acceptability curves.[61]

Process evaluation and patient experience

We plan to use a qualitative study design to describe the barriers and facilitators to implementation of the AGCH concept and describe the experiences of the patients and healthcare professionals with the AGCH. We will conduct semi-structured interviews with various stakeholders, such as geriatricians, nurses, physiotherapists and hospital administrators. These interviews will concern the implementation of the AGCH concept. In addition, semi-structured interviews with patients and informal caregivers will be conducted in order to describe the patient experience and satisfaction with this new form of care. A representative sample of patients and/or caregivers who participate in

For peer review only - http://bmjopen.bmj.com/site/about/guidelines.xhtml

the prospective cohort study will be approached and invited to be interviewed shortly after discharge from the AGCH. Stakeholders and healthcare professionals will be selected by a researcher and will be invited for an interview to discuss their experiences and opinions on the AGCH. Interviews will be typed verbatim and analysed independently by two researchers using thematic analyses.[62] In our analysis of the barriers and facilitators to implementation, we will describe these factors at three different levels: micro (healthcare professionals), meso (care organizations) and macro (legal and financial framework).[63] The findings will be summarized in matrices with the facilitators and barriers at these three different levels and can be used to develop a guideline for implementation of the AGCH elsewhere.[64]

Preliminary results

Between February 1st and December 20th, 2019, there were 362 consecutive admissions to the AGCH. Of these admissions, 26 were readmissions of patients who were already study participants. Of the remaining 336 admissions, 90 were by patients who did not meet the inclusion criteria. The remaining 246 patients or legal representatives and healthcare-proxy were approached for participation; 212 consented to participation (figure 2). The healthcare–proxy provided informed consent in 62 (29.2 %) of cases. Sixteen patients did not consent to follow-up by telephone but did consent to medical record review. The total study sample as of December 20th, 2019, consisted of 212 participants at baseline. Table 2 displays the baseline characteristics of this group. Participants had a mean age (standard deviation) of 81.8 (8.4) years and 47.6 % were male. Most participants were living independently before admission (81.1%). The most frequent admission diagnoses were infectious diseases (28.3%, mostly urinary tract infections), respiratory-related diseases (25.5%, of which half were pneumonia), and other (geriatric) diagnoses such as falls, delirium or sudden unexplained functional decline (30.2%). The main cardiovascular (9.4%) admission diagnosis was

For peer review only - http://bmjopen.bmj.com/site/about/guidelines.xhtml

exacerbation of heart failure. The median (interquartile range) length of stay was 8.0 days (5.0-12.0), and 83.7 % of patients were discharged to their original living situation.

Discussion

The complex acute medical needs of older patients require the delivery of specialized geriatric care. The traditional hospital environment may however not support recovery and maintaining independence. The AGCH aims to deliver care that focuses on medical treatment, early rehabilitation and proper transitions of care for older adults with multiple chronic conditions.[29,65] The AGCH is unique in the Netherlands in its aim to combine multiple evidenced-based components of care for frail older persons at an alternative location for hospital care. The proposed research will provide insight into the clinical and economic effectiveness of care delivered at the AGCH, compared to hospital care.

Our preliminary results show that data collection at the AGCH is feasible and we expect to recruit enough patients to evaluate the primary outcome. There are also limitations to the design of this study. It is a non-randomized study and historic cohorts are used as control groups. Therefore, baseline differences between the intervention and control groups may hamper the matching between the groups. Additionally, the data from the historic cohorts were not collected in the same time period as the AGCH cohort. This is a limitation as work processes in hospitals may have changed over the years, which could influence our results. However, the two control populations do represent a geriatric population that was admitted for exacerbations of chronic conditions and acute illnesses that frequently occur in older persons. The strengths of the study are the involvement of patients and informal caregivers in the design of the concept of the AGCH. Moreover, a process evaluation will address the barriers and facilitators to implementation of the AGCH in the Dutch Healthcare

For peer review only

system. In short, this research will provide valuable insights into the implementation of this concept of care in other regions of the Netherlands and abroad.

Ethics and Dissemination

This study will be carried out in accordance with the Declaration of Helsinki and current ethical requirements. The outcomes of this study will be reported according to the STROBE guidelines for cohort studies.[30] This study will evaluate both the effectiveness of this type of care delivery and the costs that are involved, allowing for the system to be implementation elsewhere. The findings of this study will be published in peer-reviewed journals.

Acknowledgements
The authors thank all the (care) professionals from the Amsterdam University Medical Centres, Cordaan and Zilveren Kruis who have worked on the development of the AGCH. Thank you for your time, advice and cooperation. We would also like to thank the members of the AGCH study group, which are the clinicians who work at the Geriatrics Department of the Amsterdam University Medical Centres and who support the data collection at the AGCH.

Author contributions
MER, JMV and RS contributed to the design of the protocol and drafting of the manuscript. IO and BMB made substantial contributions to the design and clinical aspects of the protocol. BMB conceived the study and wrote the funding applications. All authors critically revised the manuscript and approved the final version of this manuscript.

Funding statement
This research received funding though ZonMw, the Netherlands Organization for Health Research and Development, project number 808393598041. The care provided at the AGCH (WijkKliniek) is provided in a partnership between Cordaan, a community and home-care organization, and the Amsterdam University Medical Centres, location Academic Medical Centre. The AGCH (WijkKliniek) is financially supported by Zilveren Kruis, a health insurance company.

Patient consent
Not required.

Competing interests statement- None Declared

Ethics approval
Based on the study protocol, the Ethics Committee (METC) of the Amsterdam University Medical Centre waived the obligation for the study to undergo formal ethical approval as is described under

For peer review only

For peer review only - http://bmjopen.bmj.com/site/about/guidelines.xhtml

Dutch law in the Medical Research in Humans Act, January 2019 (ref W17_474 # 19.001). As this is a prospective study and pseudonymised data is used, written informed consent was obtained from the participants prior to participation. This is in line with current European legislation under the General Data Protection Regulation (GDPR).

References

1. McCabe JJ, Kennelly SP. Acute care of older patients in the emergency department: strategies to improve patient outcomes. *Open Access Emerg Med.* 2015;7:45-54.
2. Centraal Bureau voor de Statistiek. Ziekenhuisopnamen; kerncijfers; geslacht, leeftijd en regio. http://statline.cbs.nl/StatWeb/publication/?DM=SLNL&PA=71857ned. Published 2014. Accessed.
3. Government of the Netherlands Ministry of Health Welfare and Sport- Living Independently for Longer https://www.government.nl/topics/care-and-support-at-home/living-independently-for-longer. Published 2019. Accessed.
4. Jayaprakash N, O'Sullivan R, Bey T, Ahmed SS, Lotfipour S. Crowding and delivery of healthcare in emergency departments: the European perspective. *West J Emerg Med.* 2009;10(4):233-239.
5. de Kousemaeker G, Vandermeulen L. *Ontwikkelingen Langdurige Zorg* Fluent Healthcare Amsterdam 2017.
6. DeFrances CJ, Lucas CA, Buie VC, Golosinskiy A. 2006 National Hospital Discharge Survey. *National health statistics reports.* 2008(5):1-20.
7. Boyd CM, Xue QL, Guralnik JM, Fried LP. Hospitalization and development of dependence in activities of daily living in a cohort of disabled older women: the Women's Health and Aging Study I. *The journals of gerontology Series A, Biological sciences and medical sciences.* 2005;60(7):888-893.
8. Buurman BM, Han L, Murphy TE, et al. Trajectories of Disability Among Older Persons Before and After a Hospitalization Leading to a Skilled Nursing Facility Admission. *Journal of the American Medical Directors Association.* 2016;17(3):225-231.
9. Fogg C, Griffiths P, Meredith P, Bridges J. Hospital outcomes of older people with cognitive impairment: An integrative review. *International journal of geriatric psychiatry.* 2018.
10. van Seben R, Reichardt LA, Aarden J, et al. Geriatric syndromes from admission to 3 months post-discharge and their association with recovery. *Innovation in Aging.* 2017;1(suppl_1):902-903.
11. Boyd CM, Landefeld CS, Counsell SR, et al. Recovery of activities of daily living in older adults after hospitalization for acute medical illness. *Journal of the American Geriatrics Society.* 2008;56(12):2171-2179.
12. Kansagara D, Englander H, Salanitro A, et al. Risk prediction models for hospital readmission: a systematic review. *Jama.* 2011;306(15):1688-1698.
13. Brown CJ, Redden DT, Flood KL, Allman RM. The underrecognized epidemic of low mobility during hospitalization of older adults. *Journal of the American Geriatrics Society.* 2009;57(9):1660-1665.
14. Agmon M, Zisberg A, Gil E, Rand D, Gur-Yaish N, Azriel M. Association Between 900 Steps a Day and Functional Decline in Older Hospitalized Patients. *JAMA internal medicine.* 2017;177(2):272-274.
15. Kortebein P, Symons TB, Ferrando A, et al. Functional impact of 10 days of bed rest in healthy older adults. *The journals of gerontology Series A, Biological sciences and medical sciences.* 2008;63(10):1076-1081.
16. Zisberg A, Shadmi E, Gur-Yaish N, Tonkikh O, Sinoff G. Hospital-associated functional decline: the role of hospitalization processes beyond individual risk factors. *Journal of the American Geriatrics Society.* 2015;63(1):55-62.

For peer review only

For peer review only - http://bmjopen.bmj.com/site/about/guidelines.xhtml

17. Missildine K, Bergstrom N, Meininger J, Richards K, Foreman MD. Sleep in hospitalized elders: a pilot study. *Geriatric nursing (New York, NY).* 2010;31(4):263-271.

18. Missildine K, Bergstrom N, Meininger J, Richards K, Foreman MD. Case studies: is the sleep of hospitalized elders related to delirium? *Medsurg Nurs.* 2010;19(1):39-46.

19. Dasgupta M, Brymer C. Poor functional recovery after delirium is associated with other geriatric syndromes and additional illnesses. *International psychogeriatrics.* 2015;27(5):793-802.

20. Rodakowski J, Rocco PB, Ortiz M, et al. Caregiver Integration During Discharge Planning for Older Adults to Reduce Resource Use: A Meta-analysis. *Journal of the American Geriatrics Society.* 2017.

21. Holland DE, Mistiaen P, Bowles KH. Problems and unmet needs of patients discharged "home to self-care". *Professional case management.* 2011;16(5):240-250; quiz 251-242.

22. Naylor MD, Brooten D, Campbell R, et al. Comprehensive discharge planning and home follow-up of hospitalized elders: a randomized clinical trial. *Jama.* 1999;281(7):613-620.

23. Mechanic R. Post-acute care--the next frontier for controlling Medicare spending. *The New England journal of medicine.* 2014;370(8):692-694.

24. Westphal EC, Alkema G, Seidel R, Chernof B. How to Get Better Care with Lower Costs? See the Person, Not the Patient. *Journal of the American Geriatrics Society.* 2016;64(1):19-21.

25. Conley J, O'Brien CW, Leff BA, Bolen S, Zulman D. Alternative Strategies to Inpatient Hospitalization for Acute Medical Conditions: A Systematic Review. *JAMA internal medicine.* 2016;176(11):1693-1702.

26. Griffiths PD, Edwards MH, Forbes A, Harris RL, Ritchie G. Effectiveness of intermediate care in nursing-led in-patient units. *The Cochrane database of systematic reviews.* 2007(2):CD002214.

27. Inzitari M, Gual N, Roig T, et al. Geriatric Screening Tools to Select Older Adults Susceptible for Direct Transfer From the Emergency Department to Subacute Intermediate-Care Hospitalization. *Journal of the American Medical Directors Association.* 2015;16(10):837-841.

28. Stadindex. Stadindex- Noord- Holland https://www.stadindex.nl/noord-holland. Published 2019. Accessed 11th of June 2019

29. Stuck AE. [Multidimensional geriatric assessment in the acute hospital and ambulatory practice]. *Schweizerische medizinische Wochenschrift.* 1997;127(43):1781-1788.

30. von Elm E, Altman DG, Egger M, et al. The Strengthening the Reporting of Observational Studies in Epidemiology (STROBE) statement: guidelines for reporting observational studies. *Journal of clinical epidemiology.* 2008;61(4):344-349.

31. Folstein MF, Folstein SE, McHugh PR. "Mini-mental state". A practical method for grading the cognitive state of patients for the clinician. *Journal of psychiatric research.* 1975;12(3):189-198.

32. Buurman BM, Parlevliet JL, Allore HG, et al. Comprehensive Geriatric Assessment and Transitional Care in Acutely Hospitalized Patients: The Transitional Care Bridge Randomized Clinical Trial. *JAMA internal medicine.* 2016;176(3):302-309.

33. Buurman BM, Parlevliet JL, van Deelen BA, de Haan RJ, de Rooij SE. A randomised clinical trial on a comprehensive geriatric assessment and intensive home follow-up after hospital discharge: the Transitional Care Bridge. *BMC health services research.* 2010;10:296.

34. Reichardt LA, Aarden JJ, van Seben R, et al. Unravelling the potential mechanisms behind hospitalization-associated disability in older patients; the Hospital-Associated Disability and impact on daily Life (Hospital-ADL) cohort study protocol. *BMC geriatrics.* 2016;16(1):59.

35. Katz S. Assessing self-maintenance: activities of daily living, mobility, and instrumental activities of daily living. *Journal of the American Geriatrics Society.* 1983;31(12):721-727.

36. EuroQol--a new facility for the measurement of health-related quality of life. *Health policy (Amsterdam, Netherlands).* 1990;16(3):199-208.

For peer review only

For peer review only - http://bmjopen.bmj.com/site/about/guidelines.xhtml

37. Ellis G, Whitehead MA, Robinson D, O'Neill D, Langhorne P. Comprehensive geriatric assessment for older adults admitted to hospital: meta-analysis of randomised controlled trials. *BMJ (Clinical research ed).* 2011;343:d6553.
38. Charlson ME, Pompei P, Ales KL, MacKenzie CR. A new method of classifying prognostic comorbidity in longitudinal studies: development and validation. *Journal of chronic diseases.* 1987;40(5):373-383.
39. Inouye SK, van Dyck CH, Alessi CA, Balkin S, Siegal AP, Horwitz RI. Clarifying confusion: the confusion assessment method. A new method for detection of delirium. *Annals of internal medicine.* 1990;113(12):941-948.
40. NVKG-Dutch-Society-for-Clinical-Geriatrics. Meetinstrumenten voor diagnose delier. https://richtlijnendatabase.nl/richtlijn/delier_bij_volwassenen/meetinstrumenten_voor_diagnose_delier.html#onderbouwing. Published 2013. Accessed 8th of January 2019.
41. Heim N, van Fenema EM, Weverling-Rijnsburger AW, et al. Optimal screening for increased risk for adverse outcomes in hospitalised older adults. *Age and ageing.* 2015;44(2):239-244.
42. Schuurmans MJ, Shortridge-Baggett LM, Duursma SA. The Delirium Observation Screening Scale: a screening instrument for delirium. *Res Theory Nurs Pract.* 2003;17(1):31-50.
43. van der Mast RC, Vinkers DJ, Stek ML, et al. Vascular disease and apathy in old age. The Leiden 85-Plus Study. *International journal of geriatric psychiatry.* 2008;23(3):266-271.
44. Hoogerduijn JG, Schuurmans MJ, Duijnstee MS, de Rooij SE, Grypdonck MF. A systematic review of predictors and screening instruments to identify older hospitalized patients at risk for functional decline. *Journal of clinical nursing.* 2007;16(1):46-57.
45. Katz S, Downs TD, Cash HR, Grotz RC. Progress in development of the index of ADL. *The Gerontologist.* 1970;10(1):20-30.
46. Laan W, Zuithoff NP, Drubbel I, et al. Validity and reliability of the Katz-15 scale to measure unfavorable health outcomes in community-dwelling older people. *The journal of nutrition, health & aging.* 2014;18(9):848-854.
47. Stichting-effectieve-ouderenzorg. Comprehensive Geriatric Assesment TZB https://effectieveouderenzorg.nl/zorgmodellen/comprehensive-geriatric-assessment/. Published 2013. Accessed 8th of January 2019.
48. Roberts HC, Denison HJ, Martin HJ, et al. A review of the measurement of grip strength in clinical and epidemiological studies: towards a standardised approach. *Age and ageing.* 2011;40(4):423-429.
49. Guralnik JM, Simonsick EM, Ferrucci L, et al. A short physical performance battery assessing lower extremity function: association with self-reported disability and prediction of mortality and nursing home admission. *Journal of gerontology.* 1994;49(2):M85-94.
50. McCaffery M, Beebe A. Pain: clinical manual for nursing practice. *CV Mosby: St Louis.* 1989.
51. Hwang SS, Chang VT, Cogswell J, Kasimis BS. Clinical relevance of fatigue levels in cancer patients at a Veterans Administration Medical Center. *Cancer.* 2002;94(9):2481-2489.
52. Kruizenga HM, Seidell JC, de Vet HC, Wierdsma NJ, van Bokhorst-de van der Schueren MA. Development and validation of a hospital screening tool for malnutrition: the short nutritional assessment questionnaire (SNAQ). *Clinical nutrition (Edinburgh, Scotland).* 2005;24(1):75-82.
53. Likert. A technique for the measurement of attitudes. In. Archives of Psychology, 140, 1–55.1932.
54. Twisk JW. *Introduction to applied biostatistics (Dutch)* 3ed. Amsterdam: Reed Business Education; 2017.
55. White IR, Royston P, Wood AM. Multiple imputation using chained equations: Issues and guidance for practice. *Stat Med.* 2011;30(4):377-399.
56. van Walraven C, Seth R, Austin PC, Laupacis A. Effect of discharge summary availability during post-discharge visits on hospital readmission. *Journal of general internal medicine.* 2002;17(3):186-192.

For peer review only - http://bmjopen.bmj.com/site/about/guidelines.xhtml

57. Benjamini Y. HY. Controlling the false discovery rate: a pratical and powerful approach to multiple testing. . *Journal of the Royal Statistical Society* 1995;57(1):289-300.

58. Hochberg Y. A sharper Bonferroni procedure of multiple tests of significance In. Vol 4. 75 ed. Biometrika 1988:800-802.

59. Hakkaart-van Roijen L V-d-LN, Bouwmans C, Kanters C, Tan SS. Dutch manual for costing in economic evaluations. In. Diemen National Health Care Institute 2015.

60. Lamers LM, Stalmeier PF, McDonnell J, Krabbe PF, van Busschbach JJ. [Measuring the quality of life in economic evaluations: the Dutch EQ-5D tariff]. *Nederlands tijdschrift voor geneeskunde.* 2005;149(28):1574-1578.

61. Willan AR BA. *Statistical analysis of cost-effectiveness data.* John Wiley and Sons 2006.

62. Braun V, Clarke V. Using thematic analysis in psychology. *Qualitative research in psychology.* 2006;3(2):77-101.

63. Meiland FJ, Droes RM, De Lange J, Vernooij-Dassen MJ. Development of a theoretical model for tracing facilitators and barriers in adaptive implementation of innovative practices in dementia care. *Archives of gerontology and geriatrics Supplement.* 2004(9):279-290.

64. Moore GF, Audrey S, Barker M, et al. Process evaluation of complex interventions: Medical Research Council guidance. *BMJ (Clinical research ed).* 2015;350:h1258.

65. van Seben R, Geerlings SE, Buurman BM. [10 tips for improving patient handovers from hospital to other health care settings]. *Nederlands tijdschrift voor geneeskunde.* 2017;161(0):D1031.

For peer review only

For peer review only - http://bmjopen.bmj.com/site/about/guidelines.xhtml

Figure 1 Patient admission process and criteria, components of the AGCH intervention and goals.

(uploaded separately as an image)

CGA= Comprehensive Geriatric Assessment[29]
GP= General Practitioner

For peer review only

For peer review only - http://bmjopen.bmj.com/site/about/guidelines.xhtml

Figure 2 Diagram of patient participation between February 1st and December 20th, 2019.

(uploaded separately as an image)

For peer review only

For peer review only - http://bmjopen.bmj.com/site/about/guidelines.xhtml

Table 1 Overview of the content and description of the (outcome) measurements and timing of the measurements at the Acute Geriatric Community Hospital.

	**Description and/or instrument**	**H1**	**H2**	**P1**	**P3**	**P6**
1. Medical and demographical data						
**Sociodemographic data**	Date of birth, age at admission, sex, level of education, living conditions, marital status	R				
**Data on admission**	Time spent at the ED, admission diagnosis, date and time of admission	R				
**Chronic conditions**	Charlson Comorbidity Index[38]	R				
**Polypharmacy**	Number of drugs	R				
**Mortality**	Date of death		R	R	R	R
2. Cognitive functioning						
**Cognitive impairment**	Mini-Mental State Examination (MMSE)[31]	R				
**Delirium**	Safety management system patient screening (VMS)[41]<br>Confusion Assessment Method (CAM)[39]<br>Delirium Observation Scale (DOS)[42]	N/D	N/D			
3. Psychosocial functioning and quality of life						
**Apathy**	Geriatric Depression Scale (GDS-3)[43]	N	R	R	R	R
**Social network and informal care**	Presence and frequency of informal care	R		R	R	R
**Quality of life and health status**	EQ-5D[36]	R		R	R	R
4. Physical functioning						
**Identifying at-risk-patients**	ISAR-HP- Identifying Seniors at Risk score[44]	N				
**Functional status**	Activities of daily Living (ADL) modified Katz-ADL score[35]	N				
**(Im)mobility**	Using a walking aid, information from the Katz-ADL questions on exercise	N				
**Handgrip strength**	Jamar[48]	P				
**Gait speed**	Short Physical Performance Battery (SPPB)[49]	P				
**Falling**	Fall history<br>Falls in the AGCH<br>Numeric Rating Scale (NRS) on the fear of falling[34]	N<br><br>N	R<br><br>R	R<br><br>R	R<br><br>R	R<br><br>R
**Pain**	Numeric Rating Scale (NRS) on pain[50]	N	R	R	R	R
**Fatigue**	Numeric Rating Scale (NRS) on fatigue [51]	N	R	R	R	R
**Nutrition**	Short Nutritional Assessment Questionnaire (SNAQ)[52]	N				
5. Healthcare utilization and satisfaction with care						
**Medical care during admission**	Diagnostics performed in the AGCH<br>Readmission to university hospital<br>Length of stay at the AGCH		R			

For peer review only

For peer review only - http://bmjopen.bmj.com/site/about/guidelines.xhtml

**Hospital readmission**	Readmission rate to the hospital or AGCH		R	R	R	R
**Health care utilization**	Home care, medical specialist care, temporary institutional care, primary care	R		R	R	R
**Satisfaction with Care**	Eight question questionnaire[53]		R	(R)*		

H1= at admission, H2= at discharge, P1= one month after discharge, P3 = three months after discharge, P6 = six months after discharge. N=nurse Geriatric Community Care Hospital, P= physiotherapist, D= Doctor/attending physician, R= researcher/research nurse. *in case the assessment was missed at H2.

For peer review only

Table 2 Baseline characteristics of the study participants

**Variable**	**N=212**
**Age in years,** mean (SD)	81.8 (8.4)
**Male,** N (%)	101 (47.6)
**Living arrangements before admission,** N (%)	
Independent	172 (81.1)
Assisted living/senior residence	31 (14.6)
Nursing home/other	9 (4.2)
**Marital status,** N **(%)**	
Widow/widower	94 (44.5)
Married or living together	71 (33.6)
Single or divorced	46 (21.8)
**Education,** N (%)	
Primary school	36 (18.7)
Elementary technical/domestic science school	41 (21.2)
Secondary vocational education	65 (33.7)
Higher level high school/third-level education	51 (26.4)
**Born in the Netherlands,** N (%)	158 (76.0)
**Katz-ADL (6 item) score[a] upon admission,** median (IQR)	3.0 (1.0-5.0)
**MMSE score[b],** mean (SD)	23.7 (4.7)
**Polypharmacy[c],** N (%)	159 (75.0)
**Hospitalization in past 6 months,** N (%)	61 (31.1)
**Charlson Comorbidity Index[d],** mean (SD)	2.8 (2.0)
**Primary admission diagnosis,** N (%)	
Infectious diseases	60 (28.3)
Respiratory (including pneumonia)	54 (25.5)
Gastrointestinal	9 (4.2)
Cardiovascular	20 (9.4)
Neurologic	16 (7.5)
Other (e.g., falls, delirium, sudden unexplained functional decline)	53 (30.2)

SD, standard deviation; IQR, interquartile range
[a]Score ranging from 0-6, with a higher score indicating more dependence in activities of daily living[35]
[b]Score ranging from 0-30, with a score of ≤23 indicating possible cognitive impairment [31]
[c]Use of 5 drugs or more
[d]Ranging from 0-31, with a higher score indicating more severe comorbidity[38]

For peer review only - http://bmjopen.bmj.com/site/about/guidelines.xhtml

For peer review only

For peer review only - http://bmjopen.bmj.com/site/about/guidelines.xhtml

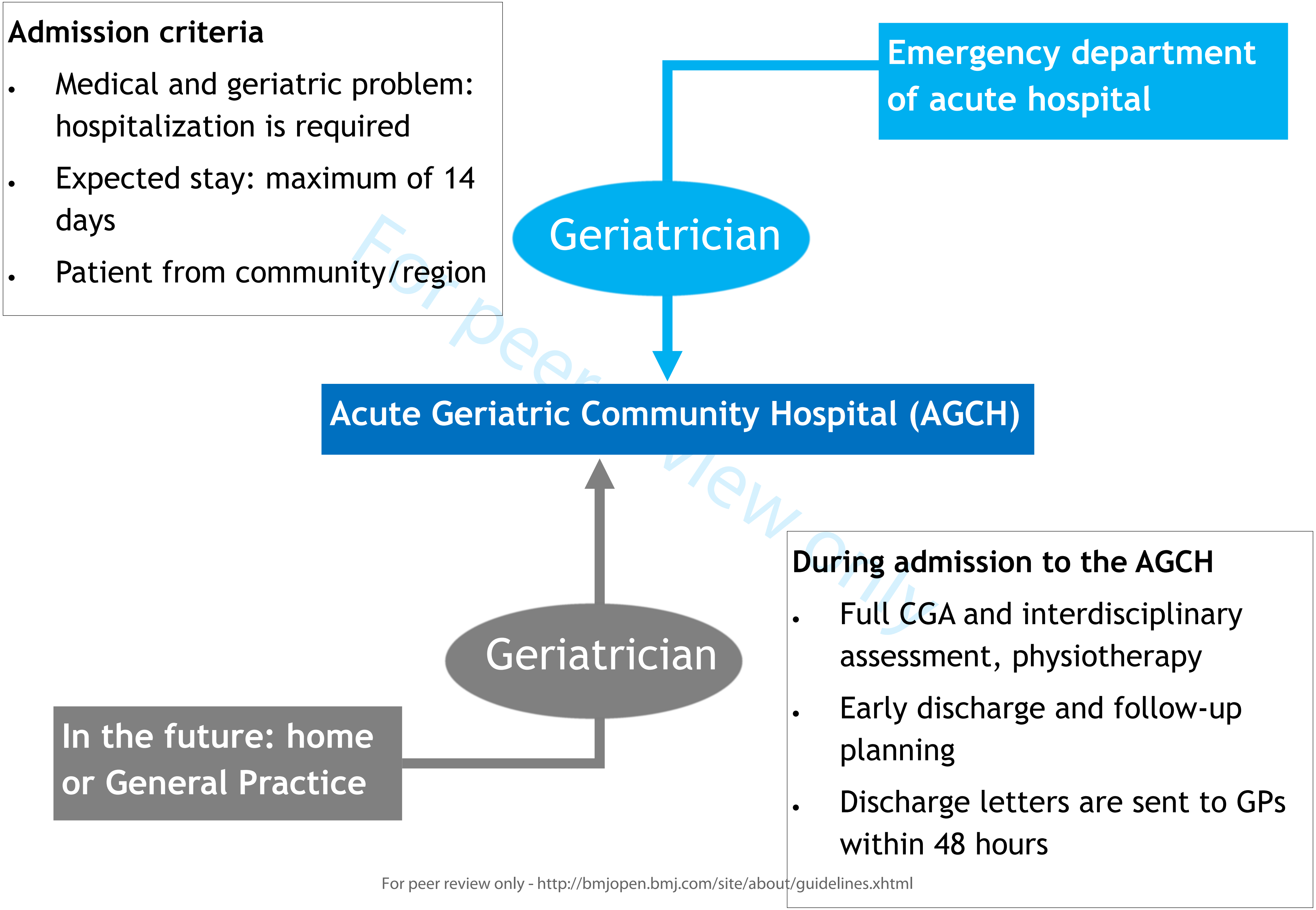

For peer review only

Figure 2 Diagram of patient participation between February 1st and December 20th 2019.

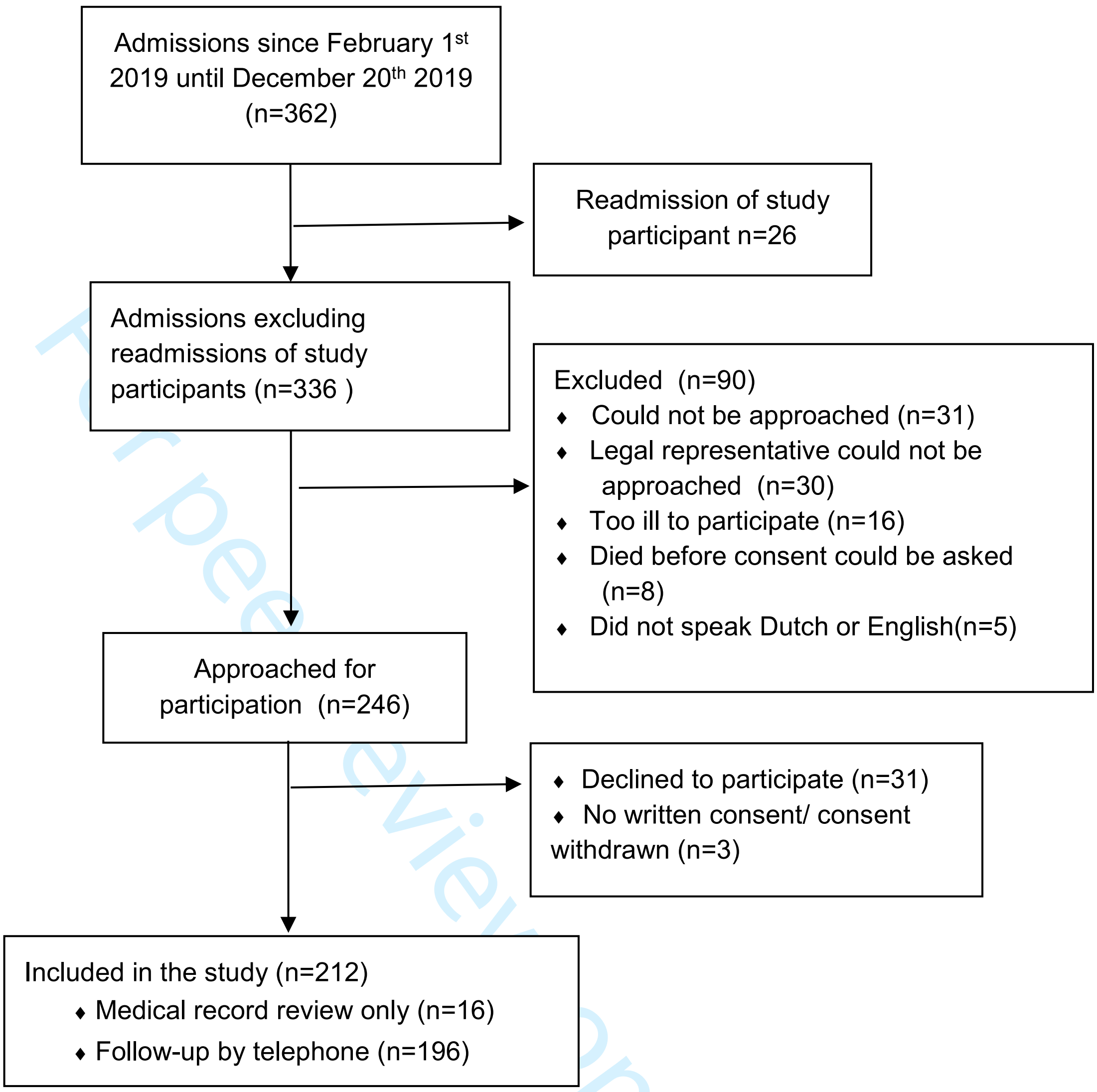

For peer review only

Supplementary table Overview of the content and description of (outcome) measurements and timing of measurements at the Acute Geriatric Community Hospital compared to the measurements available in the two control groups.

	**Description and/or instrument**	**H1**	**H2**	**P1**	**P3**	**P6**
1. Medical and demographical data						
**Sociodemographic data**	Date of birth, age at admission, sex, level of education, living conditions, marital status	T A				
**Data on admission**	Time spent at the ED*, admission diagnosis, date and time of admission	T A				
**Chronic conditions**	Charlson Comorbidity Index[38]	T A				
**Polypharmacy**	Number of drugs	T A				
**Mortality**	Date of death		T A			
2. Cognitive functioning						
**Cognitive impairment**	Mini Mental State Examination (MMSE)	T A				
**Delirium**	Safety management system patient screening (VMS)[41]<br>Confusion Assessment Method (CAM)[39]<br>Delirium Observation Scale (DOS)[42]	T<br>TA†<br>T†	 <br>T			
3. Psychosocial functioning and quality of life						
**Apathy**	Geriatric Depression Scale (GDS-3)[43]	A	A	A	A	
**Social network and informal care**	Presence and frequency of informal care	T		-	-	T
**Quality of life and health status**	EQ-5D[36]	T A		A	A	T A
4. Physical functioning						
**Identifying at-risk-patients**	ISAR-HP- Identifying Seniors at Risk score[44]	T				
**Functional status**	Activities of daily Living (ADL) modified Katz-ADL score[35]	T A	A	A	A	T
**(Im)mobility**	Using walking aid, information from the Katz-ADL questions on exercise	T A				
**Handgrip strength**	Jamar[48]	T A	A			
**Gait speed**	Short Physical Performance Battery SPPB[49]	T A	A			
**Falling**	Fall history<br>Falls in the AGCH<br>Numeric Rating Scale (NRS) on the fear of falling[34]	T A<br>n/a<br>A	-<br>n/a<br>A	A<br>n/a<br>A	A<br>n/a<br>A	T<br>n/a<br>-
**Pain**	Numeric Rating Scale (NRS) on pain[50]	T A	A	A	A	-
**Fatigue**	Numeric Rating Scale (NRS) on fatigue[51]	T A	A	A	A	-
**Nutrition**	Short Nutritional Assessment Questionnaire (SNAQ)[52]	T A				
5. Healthcare utilization and satisfaction with care						
**Medical care during admission**	Diagnostics performed in the AGCH<br>Readmission to university hospital		n/a			

For peer review only

	Length of stay at the AGCH					
**Hospital readmission**	Readmission rate to the hospital or AGCH			A	T A	T
**Health care utilization**	Home care, medical specialist care, temporary institutional care, primary care.	T		A	A	T
**Satisfaction with Care**	Eight question questionnaire[53]		-	-		

Grey tone= measurement in prospective cohort study at the AGCH.

H1= at admission, H2= at discharge, P1= one month after discharge, P3 = three months after discharge, P6 = six months after discharge.

T= available from *Transitional Care Bridge study(TCB)*[1]

A= available from *Hospital- ADL study(H-ADL)*[2]

** and* - =Not available from TCB or H-ADL

†=Single baseline measurement

n.a.= not applicable

For peer review only

Appendix 1 STROBE statement checklist

STROBE Statement—Checklist of items that should be included in reports of ***cohort studies***

	Item No	Recommendation	Item found on page
**Title and abstract**	1	(*a*) Indicate the study's design with a commonly used term in the title or the abstract	1 and 3
		(*b*) Provide in the abstract an informative and balanced summary of what was done and what was found	Page 3
**Introduction**			
Background/rationale	2	Explain the scientific background and rationale for the investigation being reported	Page 5-7
Objectives	3	State specific objectives, including any prespecified hypotheses	Page 7
**Methods**			
Study design	4	Present key elements of study design early in the paper	Page 8 -9
Setting	5	Describe the setting, locations, and relevant dates, including periods of recruitment, exposure, follow-up, and data collection	Page 7-15
Participants	6	(*a*) Give the eligibility criteria, and the sources and methods of selection of participants. Describe methods of follow-up	Page 7-10 and 11
		(*b*) For matched studies, give matching criteria and number of exposed and unexposed	Page 9 and 10
Variables	7	Clearly define all outcomes, exposures, predictors, potential confounders, and effect modifiers. Give diagnostic criteria, if applicable	Page 11 – 15
Data sources/ measurement	8*	For each variable of interest, give sources of data and details of methods of assessment (measurement). Describe comparability of assessment methods if there is more than one group	Page 11-15 (supplementary table)
Bias	9	Describe any efforts to address potential sources of bias	Page 16
Study size	10	Explain how the study size was arrived at	Page 16
Quantitative variables	11	Explain how quantitative variables were handled in the analyses. If applicable, describe which groupings were chosen and why	Page 16

For peer review only

Statistical methods	12	(*a*) Describe all statistical methods, including those used to control for confounding	Page 16
		(*b*) Describe any methods used to examine subgroups and interactions	n/a
		(*c*) Explain how missing data were addressed	Page 16
		(*d*) If applicable, explain how loss to follow-up was addressed	Page 16
		(*e*) Describe any sensitivity analyses	n/a
**Results**			
Participants	13*	(a) Report numbers of individuals at each stage of study—eg numbers potentially eligible, examined for eligibility, confirmed eligible, included in the study, completing follow-up, and analysed	Page 18 and 26 (figure 2)
		(b) Give reasons for non-participation at each stage	Page 18 and 26 (figure 2)
		(c) Consider use of a flow diagram	Page 26, figure 2
Descriptive data	14*	(a) Give characteristics of study participants (eg demographic, clinical, social) and information on exposures and potential confounders	Page 18,19 and 29
		(b) Indicate number of participants with missing data for each variable of interest	n/a
		(c) Summarise follow-up time (eg, average and total amount)	n/a
Outcome data	15*	Report numbers of outcome events or summary measures over time	n/a
Main results	16	(*a*) Give unadjusted estimates and, if applicable, confounder-adjusted estimates and their precision (eg, 95% confidence interval). Make clear which confounders were adjusted for and why they were included	n/a
		(*b*) Report category boundaries when continuous variables were categorized	Page 29
		(*c*) If relevant, consider translating estimates of relative risk into absolute risk for a meaningful time period	n/a
Other analyses	17	Report other analyses done—eg analyses of subgroups and interactions, and sensitivity analyses	n/a

For peer review only

**Discussion**			
Key results	18	Summarise key results with reference to study objectives	Page 19
Limitations	19	Discuss limitations of the study, taking into account sources of potential bias or imprecision. Discuss both direction and magnitude of any potential bias	Page 19
Interpretation	20	Give a cautious overall interpretation of results considering objectives, limitations, multiplicity of analyses, results from similar studies, and other relevant evidence	Page 19
Generalisability	21	Discuss the generalisability (external validity) of the study results	n/a
**Other information**			
Funding	22	Give the source of funding and the role of the funders for the present study and, if applicable, for the original study on which the present article is based	Page 20

*Give information separately for exposed and unexposed groups.

Note: An Explanation and Elaboration article discusses each checklist item and gives methodological background and published examples of transparent reporting. The STROBE checklist is best used in conjunction with this article (freely available on the Web sites of PLoS Medicine at http://www.plosmedicine.org/, Annals of Internal Medicine at http://www.annals.org/, and Epidemiology at http://www.epidem.com/). Information on the STROBE Initiative is available at http://www.strobe-statement.org.

For peer review only
